# Autoimmune thyroiditis and comorbid autoimmune diseases: global research landscape and future directions

**DOI:** 10.3389/fimmu.2026.1746955

**Published:** 2026-07-16

**Authors:** Jie Tian, Feng Guo, Hao-Han Rao, Wan-Chen Xie, Xin-Yu Li, Huan-Wen Chen

**Affiliations:** 1 Department of Thoracic Surgery, The First Affiliated Hospital of Chongqing Medical University, Chongqing, China; 2 Department of Breast and Thyroid Surgery, Chongqing Key Laboratory of Molecular Oncology and Epigenetics, The First Affiliated Hospital of Chongqing Medical University, Chongqing, China

**Keywords:** autoimmune disease (AD), autoimmune thyroiditis (AIT), bibliometric analysis, frontier hotspots, global research trends

## Abstract

**Objective:**

Autoimmune thyroiditis (AIT) patients frequently present with coexisting autoimmune diseases (ADs), adding complexity to management and outcomes. Although this field has been extensively explored, there is a paucity of bibliometric analyses capable of clarifying worldwide research trajectories, identifying major hotspots, and outlining prospective directions.

**Methods:**

This study extracted data from PubMed, Web of Science Core Collection, and Scopus, analyzing 2,201 publications published between 1980 and 2024. Data visualization was performed using VOSviewer, CiteSpace, and the bibliometrix R package.

**Results:**

The analysis encompassed 95 countries, 2,561 institutions, and 10,355 authors, with publications across 769 sources. The United States led in the number of published articles, while the University of Pisa emerged as the most productive institution. The *Journal of Clinical Endocrinology & Metabolism* had the highest volume of publications and citations in this field. Alessandro Antonelli was identified as the most prolific author, with the highest number of co-cited authors. Keyword analysis identified five primary areas of research: immune dysregulation and inflammation mechanisms, comorbidity phenomena and shared pathological foundations, immune disturbances in infections and pregnancy, genetic susceptibility and genetic mechanisms, and biomarkers and cancer risk. Key future research areas include further exploration of precision medicine & multi-omics, microbiota’s role in immune regulation, genetic factors and polymorphisms, the relationship between comorbidities and systemic ADs, and biomarkers with causal inference.

**Conclusion:**

This study provides a comprehensive analysis of the literature on AD and AIT, offering insights into global research trends and current hotspots, while suggesting future directions for further investigation.

## 1 Introduction

Autoimmune diseases (ADs) comprise a diverse spectrum of chronic disorders characterized by dysregulated immune responses targeting self-antigens, affecting approximately 10% of the global population with rising incidence rates (1). These conditions impose significant healthcare burdens through their chronic nature, multisystem involvement, and tendency toward comorbidity clustering. Among organ-specific ADs, autoimmune thyroiditis (AIT) is the most prevalent autoimmune thyroid disease (AITD), involving immune-mediated progressive thyroid gland destruction. Hashimoto’s thyroiditis (HT) is the primary clinical manifestation of AIT (2); its global prevalence is estimated at 3-5% and has increased steadily in recent decades (3).

Pathophysiological hallmarks of AIT include thyroid-specific autoantibody production (anti-thyroid peroxidase and anti-thyroglobulin antibodies), lymphocytic infiltration, thyrocyte apoptosis, and the disruption of follicular architecture (4). Thyroid tissue damage releases autoantigenic signals that drive immune activation, primarily targeting thyroid follicular epithelial cells (TFCs). Upon recognizing pathogen-associated molecular patterns (PAMPs) and danger-associated molecular patterns (DAMPs), TFCs express Toll-like receptors, leading to pattern recognition receptor (PRR) overactivation. This cascade promotes CD4+ T cell recruitment, thyroidal infiltration, and sustained autoimmune-mediated injury (5). Clinically, AIT is the leading cause of primary hypothyroidism (6). Epidemiological evidence also associates AIT with increased cardiovascular disease risk and heightened susceptibility to certain malignancies, such as papillary thyroid carcinoma (7, 8).

A key characteristic of AIT is its frequent coexistence with various systemic ADs, a phenomenon known as polyautoimmunity that transcends single-organ pathology. Clinical observations have documented high comorbidity rates between AIT and type 1 diabetes (T1D), rheumatoid arthritis (RA), systemic lupus erythematosus (SLE), multiple sclerosis (MS), and Sjögren’s syndrome (SS) (9–13). Quantitative assessments reveal that AITD prevalence among RA patients is 1–6 times higher than in the general populations (14), while a high proportion of adult T1D cohorts develop concurrent AITD (15). Research shows that 15.7% of SS patients exhibit thyroid autoimmune inflammation, reflecting overlapping features between AIT and SS (16). In Chinese population, SLE patients have a significantly increased AITD risk, particularly those with anti-dsDNA antibodies, serositis, or hypocomplementemia (17). Similarly, MS cohorts show elevated thyroid dysfunction and higher AD prevalence, suggesting shared genetic susceptibility architectures linking AIT to various autoimmune conditions (18). These comorbidity patterns underscore AIT’s position within a broader AD spectrum, where shared immunogenetic mechanisms, environmental triggers, and dysregulated immune pathways drive multi-organ autoimmunity.

Despite growing evidence from individual studies and systematic reviews, comprehensive bibliometric analyses of the global AD-AIT research landscape remain lacking. While previous valuable bibliometric studies, such as the work by Li et al. (3), have elegantly mapped the landscape of AIT primarily as an isolated condition (focusing on localized factors like oxidative stress and specific thyroid cancers), there is a critical need to evaluate AIT through the broader lens of polyautoimmunity and shared systemic mechanisms. Bibliometric methods provide a quantitative framework for analyzing scholarly trends, citation patterns, and collaborative networks, thereby identifying research hotspots and evolutionary trajectories (19). Such analyses help identify research gaps and map international collaborations, which is essential for multidisciplinary fields like AD-AIT research.

**Figure 1:**
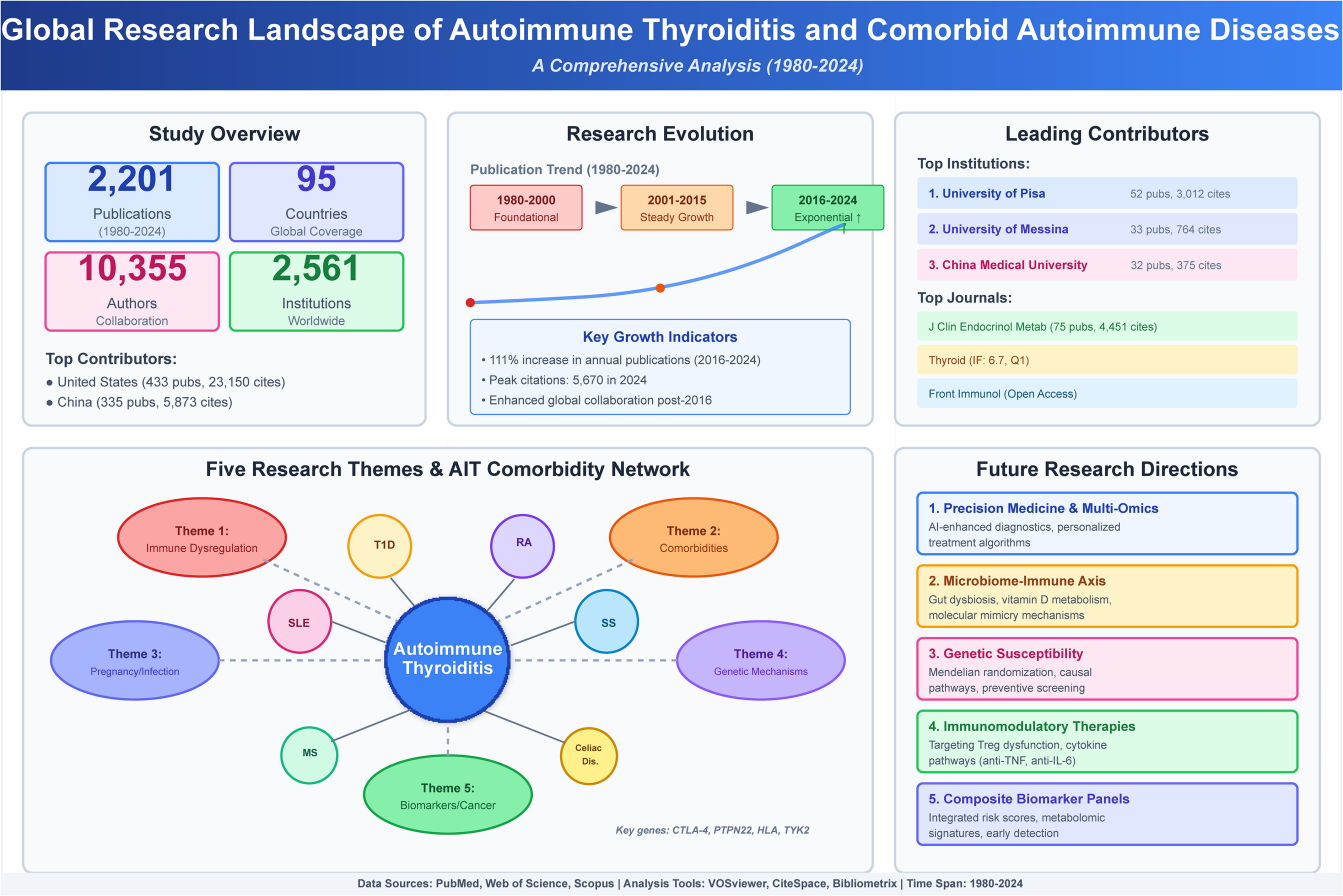
Global research landscape of autoimmune thyroiditis (AIT) and comorbid autoimmune diseases (ADs) from 1980 to 2024. The upper panels display study overview statistics, temporal research evolution across three distinct growth phases, and leading contributors. The lower left panel visualizes five interconnected research themes surrounding the central AIT node and six major comorbid conditions. The lower right panel identifies five future research priorities. Data sources: PubMed, Web of Science, Scopus; analytical platforms: VOSviewer, CiteSpace, Bibliometrix. AIT, autoimmune thyroiditis; ADs, autoimmune diseases; T1D, type 1 diabetes; RA, rheumatoid arthritis; SLE, systemic lupus erythematosus; SS, Sjögren’s syndrome; MS, multiple sclerosis; CTLA-4, cytotoxic T-lymphocyte associated protein 4; PTPN22, protein tyrosine phosphatase non-receptor type 22; HLA, human leukocyte antigen; TYK2, tyrosine kinase 2; Treg, regulatory T cell.

This study employs rigorous bibliometric approaches, integrating three authoritative databases {PubMed, Web of Science Core Collection (WoSCC), Scopus} with advanced analytical platforms to map the global research landscape of AD and AIT relationships (**Figure 1**). Our primary objectives are to: (1) quantify publication trends and growth phases; (2) assess geographic and institutional contributions; (3) identify influential journals, authors, and seminal publications; (4) determine current research hotspots through keyword clustering; and (5) project future research directions. These findings aim to provide guide future research, optimize funding allocation, and enhance the clinical understanding of AIT’s role within the AD spectrum, improving strategies for patients with polyautoimmunity. Critically, by systematically mapping research trajectories and quantifying thematic clusters, this analysis identifies which immunological mechanisms—including cytokine dysregulation, regulatory T cell (Treg) impairment, human leukocyte antigen (HLA)-linked immune tolerance breakdown, and microbiome-immune axis disruption—have attracted disproportionate investigative attention versus remaining underexplored, thereby offering translational immunologists a data-driven roadmap for prioritizing mechanistic inquiry and accelerating bench-to-bedside translation in AIT-associated polyautoimmunity.

## 2 Materials and methods

### 2.1 Data retrieval strategy and screening protocol

A comprehensive literature retrieval was executed across three authoritative bibliographic repositories: PubMed, WoSCC, and Scopus, encompassing the period from January 1, 1980, through December 31, 2024. The retrieval operation was finalized on October 31, 2025. Search algorithms targeted title and abstract fields in PubMed, while WoSCC and Scopus queries extended to title, abstract, and keyword domains. The preliminary retrieval captured 10,993 bibliographic entries distributed as follows: PubMed (n=3,326), WoSCC (n=3,565), and Scopus (n=4,102). Duplicate elimination procedures removed 7,145 redundant entries, yielding 3,848 distinct records for subsequent evaluation. A rigorous eligibility assessment was then implemented to filter document categories and thematic alignment. Topical relevance was determined by evaluating whether each publication’s primary focus substantively concerned the relationship between AIT and one or more ADs; studies in which AIT or an AD appeared only as a secondary mention incidental to a different primary research question were excluded. Publications addressing multiple concurrent ADs alongside AIT were retained in their entirety and attributed to all relevant thematic clusters during keyword and co-citation analyses, rather than being assigned to a single disease category, thereby preserving the full scope of multi-disease associations. To ensure consistency during title-and-abstract screening, two authors assessed each record independently, and disagreements were resolved by consensus with corresponding author. Entries designated as conference proceedings, correspondence, commentaries, meeting summaries, editorial content, book sections, errata, or exhibiting insufficient topical relevance were systematically excluded (n=1,647). The final analytical corpus comprised 2,201 publications, stratified into 1,857 original research contributions and 344 systematic reviews (**Supplementary Figure 1**).

### 2.2 Eligibility criteria

Inclusion criteria: (i) Publications categorized as original research investigations or review manuscripts published between 1980 and 2024; (ii) Studies demonstrably centered on AIT and ADs research domains.

Exclusion criteria: (i) Document types including proceeding paper, note, letter, meeting abstract, editorial material, book chapters, or correction; (ii) Publications exhibiting tangential or negligible relevance to AIT and ADs research, thereby guaranteeing analytical focus on methodologically sound and contextually pertinent literature.

### 2.3 Analytical instrumentation

This investigation integrated three complementary bibliometric platforms to execute multidimensional analytical procedures:

VOSviewer (version 1.6.19) was deployed for network topology generation and visualization (19, 20), specifically enabling: (i) cartographic representation of collaborative frameworks spanning national boundaries, institutional affiliations, and individual authorship; (ii) co-citation architecture analysis examining both primary publications and their reference matrices; (iii) keyword co-occurrence network development; (iv) graphical schema wherein node magnitude corresponds to publication frequency while color gradation denotes thematic classification or chronological distribution, thereby illuminating partnership dynamics and citational interdependencies.

CiteSpace (version 6.2.R3) (21, 22) facilitated: (i) dual-map overlay generation depicting cross-disciplinary citation flows; (ii) burst detection algorithms identifying emerging investigative frontiers; (iii) keyword clustering and timeline visualization to trace thematic evolution trajectories and research paradigm shifts.

Biblioshiny platform, serving as the web-accessible interface for the R-package bibliometrix (version 4.3.1) (23–25), supported: (i) cartographic visualization of global publication distribution patterns; (ii) transnational collaborative network characterization; (iii) thematic map construction and evolutionary trajectory analysis; (iv) international partnership pattern assessment.

### 2.4 Data processing and visual representation

Microsoft Office Excel 2021 was employed for quantitative metric computation, including temporal publication trajectories, citation indices, and collaboration parameters. Standardized visualization protocols were implemented uniformly across all analytical platforms to ensure graphical consistency and interpretive clarity throughout the investigation.

## 3 Results

### 3.1 Quantitative analysis of publications

**Figure 2:**
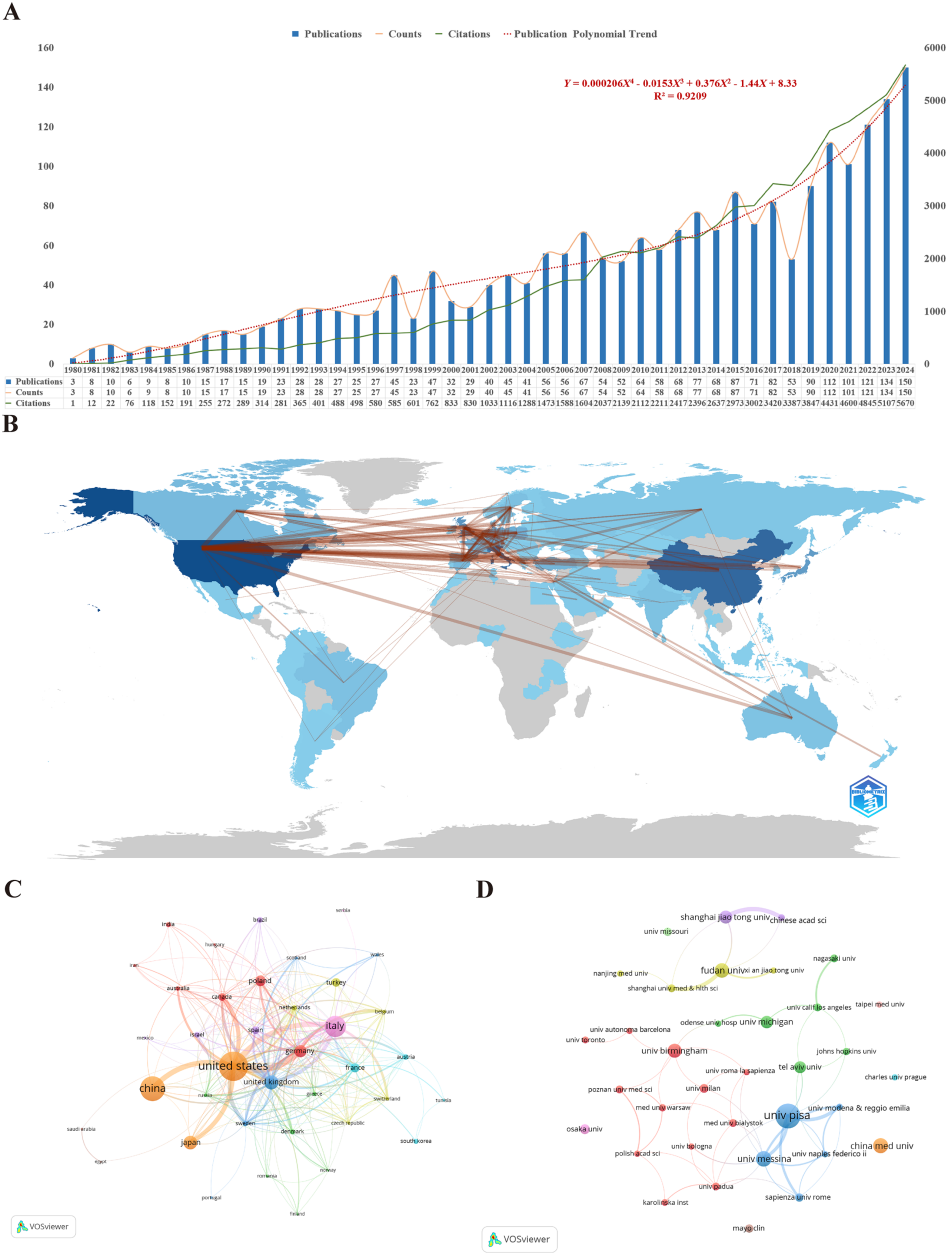
The visualization of ADs and AIT research. **(A)** Annual research output on the research of ADs and AIT. The orange curve indicates the annual publication trend. The green curve indicates the annual citation trend. The red dotted trend line is fitted to the number of publications using a quartic polynomial model. **(B)** The geographical spread of contributions. Where darker colors indicate higher publication volumes. **(C)** Cooperation network of different countries. In this network, node size reflects the number of publications per country, different colors represent distinct collaboration clusters, and line thickness indicates the strength of international collaboration. This study enrolled 38 countries based on minimum number of documents of a country equal to 10 (attraction/repulsion values: 3/-3). **(D)** The visualization of institutions on research of AD and AIT. This study selected 35 institutions based on the minimum number of publications equal to 13 for visualization, and constructed a collaborative network based on the number and relationship of publications of each institution (attraction/repulsion values: 1/-3).

The analysis included 2,201 papers from 95 countries, 2,561 organizations, and 10,355 authors, spread across 769 sources. The temporal publication trajectory spanning 1980–2024 exhibited a triphasic evolutionary pattern characterized by distinct growth dynamics (**Figure 2A**). The foundational phase (1980-2000) demonstrated minimal research activity with publications plateauing below 50 articles annually, reflecting the nascent recognition of AD-AIT as a discrete research entity. The steady expansion phase (2001-2015) witnessed gradual output escalation from 29 to 87 publications per year, coinciding with advancing immunological methodologies and molecular diagnostic capabilities. The exponential proliferation phase (2016-2024) manifested unprecedented growth acceleration, with annual publications surging from 71 to 150 articles—a 111% increase within nine years—accompanied by a dramatic citation surge peaking at 5,670 in 2024. The polynomial regression model demonstrated robust predictive validity, with the exponential trajectory particularly pronounced post-2019, potentially attributable to enhanced global research collaboration, omics technology maturation, and heightened clinical awareness of autoimmune thyroid disorders’ systemic implications.

### 3.2 Country and institution analysis

The United States demonstrated overwhelming dominance with 433 publications and 23,150 citations (average 53.46 citations/publication), followed by China (335 publications, 17.53 citations/publication) and Italy (272 publications, 44.85 citations/publication) (**Figures 2B, C**; **Table 1**). Notably, the United Kingdom exhibited the highest citation efficiency (76.37 citations/publication) despite ranking fourth in publication volume, suggesting superior research quality and international recognition. A distinct East-West dichotomy emerged, with Western nations (US, UK, Italy, Germany, France, Spain) achieving substantially higher citation rates (40.83-76.37) compared to Asian countries (China 17.53, Japan 31.87, Turkey 11.16), potentially reflecting differences in research funding infrastructure, international collaboration networks, and publication language advantages. Poland’s relatively low citation impact (18.03) despite considerable output (103 publications) mirrors patterns observed in emerging research economies. This distribution underscores that research influence in autoimmune thyroidology extends beyond mere publication quantity, with established Western medical research systems maintaining disproportionate scientific impact through enhanced visibility, collaborative frameworks, and methodological rigor.

**Table 1:** Top 10 countries and organization on the research of AD and AIT.

Rank	Country	Counts	Citations	Average citation/ publications	Organization	Counts	Citations
1	United States	433	23150	53.46	University of Pisa (Italy)	52	3012
2	China	335	5873	17.53	University of Messina (Italy)	33	764
3	Italy	272	12198	44.85	China Medical University (China)	32	375
4	United Kingdom	153	11685	76.37	Fudan University (China)	31	694
5	Japan	148	4717	31.87	University of Birmingham (United Kingdom)	30	4649
6	Germany	125	6905	55.24	Shanghai Jiao Tong University (China)	28	586
7	Poland	103	1857	18.03	University of Michigan (United States)	26	1153
8	France	80	3866	48.33	Tel Aviv University (Israel)	25	1539
9	Turkey	79	882	11.16	Osaka University (Japan)	21	509
10	Spain	64	2613	40.83	University of Milan (Italy)	19	1104

The institutional analysis revealed a predominant concentration of research productivity in European and Asian institutions, with the University of Pisa leading in publication volume (52 articles, 3,012 citations), followed by the University of Messina (33 articles) and China Medical University (32 articles) (**Figure 2D**; **Table 1**). Notably, a marked disparity emerged between publication quantity and citation impact, as exemplified by the University of Birmingham, which achieved the highest citation count (4,649) despite ranking fifth in output (30 articles), yielding a citation-per-article ratio nearly threefold higher than the leading institution. This pattern suggests a geographic stratification in research influence, with Western European and Israeli institutions (Tel Aviv University: 1,539 citations from 25 articles) demonstrating higher per-article impact compared to Asian institutions, potentially reflecting differences in research focus, methodological rigor, or established collaborative networks in the AD-AIT field.

### 3.3 Top journals and co-cited journals

**Figure 3:**
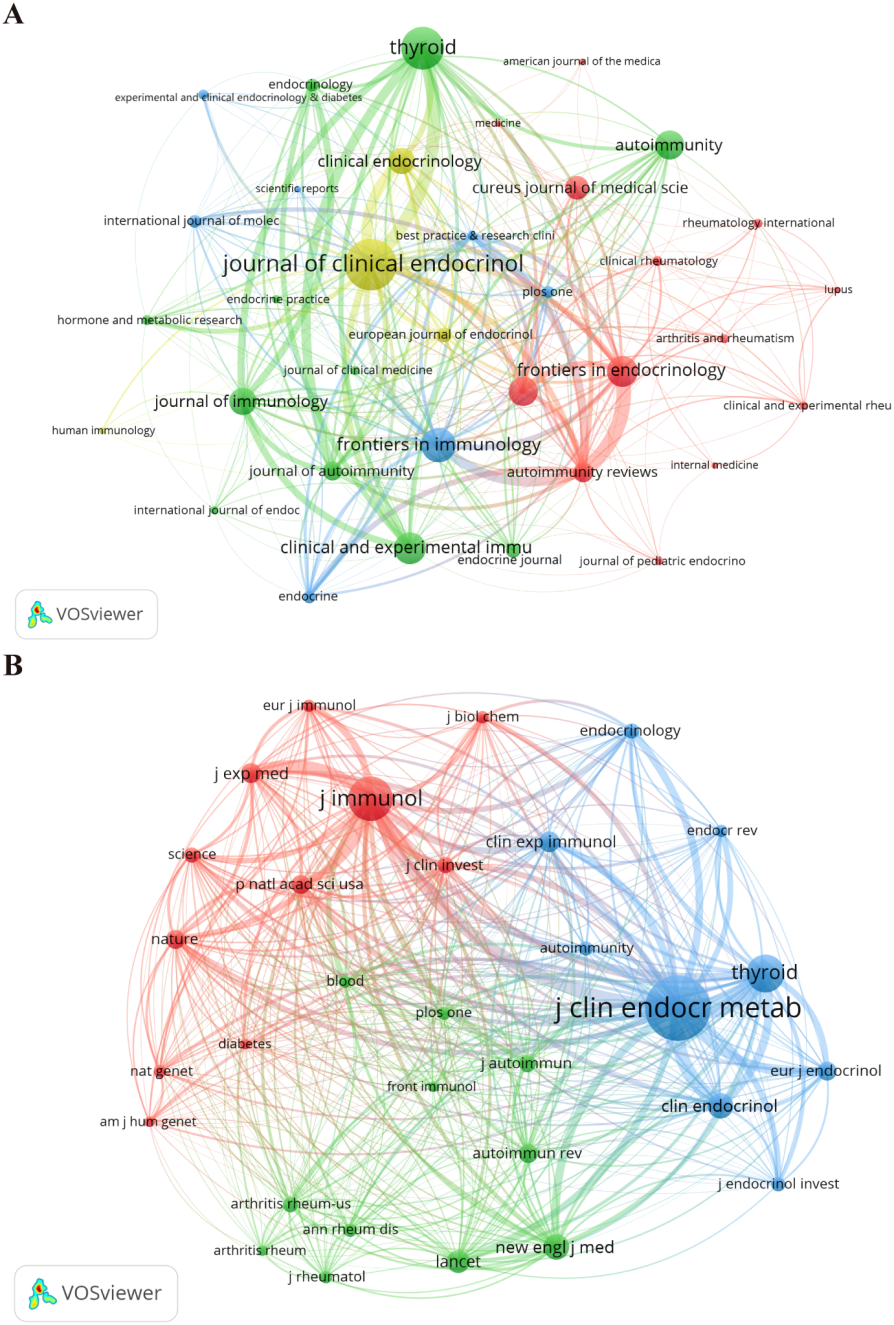
The visualization of journals **(A)** and co-cited journals **(B)** on research of on research of AD and AIT. **(A)** This study enrolled 35 journals based on the minimum number of relevant publications equal to 10 and mapped the journal network (attraction/repulsion values: 2/-1). Node size represents publication volume, colors indicate journal clusters based on research focus similarity, and line thickness shows co-citation strength between journals. **(B)** More than 500 co-citation journals (31 journals) were filtered to map the co-citation network (attraction/repulsion values:1/0). Node size reflects total co-citations, different colors represent thematic clusters, and connecting lines indicate co-citation relationships with thickness proportional to co-citation frequency.

The top 10 publishing journals demonstrated a pronounced dominance of specialized endocrinology and immunology outlets, with the *Journal of Clinical Endocrinology & Metabolism* leading in both publication volume (75 articles) and citation impact (4,451 citations), despite maintaining a moderate impact factor (IF = 5.1, Q1) (**Figure 3A**; **Table 2**). A notable discordance emerged between journal prestige and publication productivity, as evidenced by *Cureus Journal of Medical Science* ranking tenth with 34 articles yet garnering merely 76 citations (IF = 1.3, Q2), suggesting potential predatory or lower-tier publication strategies. The disciplinary composition revealed a balanced representation between endocrinology-focused journals (*Journal of Clinical Endocrinology & Metabolism*, *Thyroid*, *Clinical Endocrinology*) and immunology-oriented outlets (*Frontiers in Immunology*, *Journal of Immunology*), reflecting the interdisciplinary nature of AD-AIT research, while the prevalence of open-access platforms (*Frontiers in Immunology*, *Frontiers in Endocrinology*) indicates evolving scholarly communication patterns in this field.

**Table 2:** Top 10 journals and co-cited journals on the research of AD and AIT.

Rank	Journal	Counts	Citations	IF^a^	Q^b^	Co-cited journal	Co-citation	IF^a^	Q^b^
1	Journal of Clinical Endocrinology & Metabolism	75	4451	5.1	1	Journal of Clinical Endocrinology & Metabolism	4549	5.1	1
2	Thyroid	62	2113	6.7	1	Journal of Immunology	2891	3.4	2
3	Frontiers in Immunology	50	1554	5.9	1	Thyroid	2395	6.7	1
4	Clinical and Experimental Immunology	46	1737	3.8	2	Clinical Endocrinology	1540	2.4	3
5	Frontiers in Endocrinology	45	712	4.6	1	New England Journal of Medicine	1534	78.5	1
6	Autoimmunity	42	889	3.1	3	Lancet	1352	88.5	1
7	Journal of Endocrinological Investigation	42	945	3.5	2	Clinical and Experimental Immunology	1219	3.8	2
8	Journal of Immunology	39	2245	3.4	2	Journal of Experimental Medicine	1151	10.6	1
9	Clinical Endocrinology	38	1330	2.4	3	Nature	1106	48.5	1
10	Cureus Journal of Medical Science	34	76	1.3	2	Autoimmunity Reviews	1102	8.3	1

**^a^**The impact factor of the journal are obtained from Journal Citation Reports 2024.

**^b^**The quartile of the journal are obtained from Journal Citation Reports 2024.

The co-citation analysis unveiled a distinct hierarchical knowledge structure, wherein elite general medicine journals—*New England Journal of Medicine* (IF = 78.5), *Lancet* (IF = 88.5), and *Nature* (IF = 48.5)—occupied prominent positions despite minimal direct publication output in the dataset, collectively accumulating over 3,900 co-citations (**Figure 3B**; **Table 2**). This pattern underscores the foundational role of seminal studies published in high-impact generalist venues in shaping contemporary AITD research paradigms, while the substantial co-citation of specialized journals (*Journal of Clinical Endocrinology & Metabolism*: 4,549; *Thyroid*: 2,395) suggests an intellectual scaffold wherein field-specific empirical findings are interpreted through theoretical frameworks established by landmark publications in premier multidisciplinary outlets.

**Figure 4:**
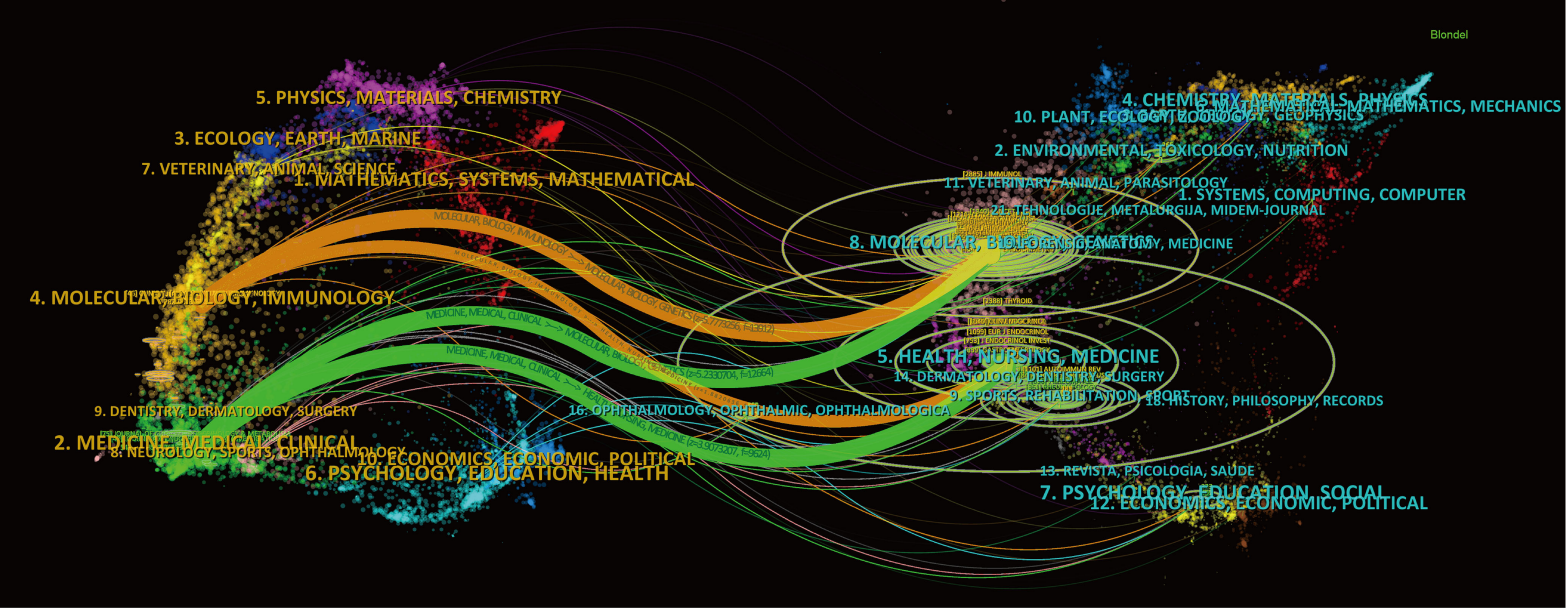
The dual-map overlay of journals on research of AD and AIT.

The dual-map overlay visualizes the bibliometric coupling between publishing venues and their referenced literature (**Figure 4**). The left-side ellipses denote journals hosting original investigations, whereas the right-side ellipses represent co-cited sources—those journals recurrently appearing together within reference lists. Citation trajectories typically originate from left-positioned journals toward right-positioned co-cited outlets, thereby mapping knowledge dissemination pathways. Two predominant citation corridors emerged: the green trajectory demonstrates substantial knowledge transfer from *Medicine/Medical/Clinical* disciplines toward *Molecular/Biology/Genetics* and *Health/Nursing/Medicine* domains; conversely, the orange trajectory reveals prominent citations originating from *Molecular/Biology/Immunology* research that reference foundational works in *Molecular/Biology/Genetics* and *Health/Nursing/Medicine* fields. This bidirectional knowledge flow confirms the translational nature of AD-AIT research, wherein mechanistic immunogenetic discoveries inform clinical practice while clinical phenotypic observations reciprocally drive molecular investigation priorities.

### 3.4 Top authors and co-cited authors

**Figure 5:**
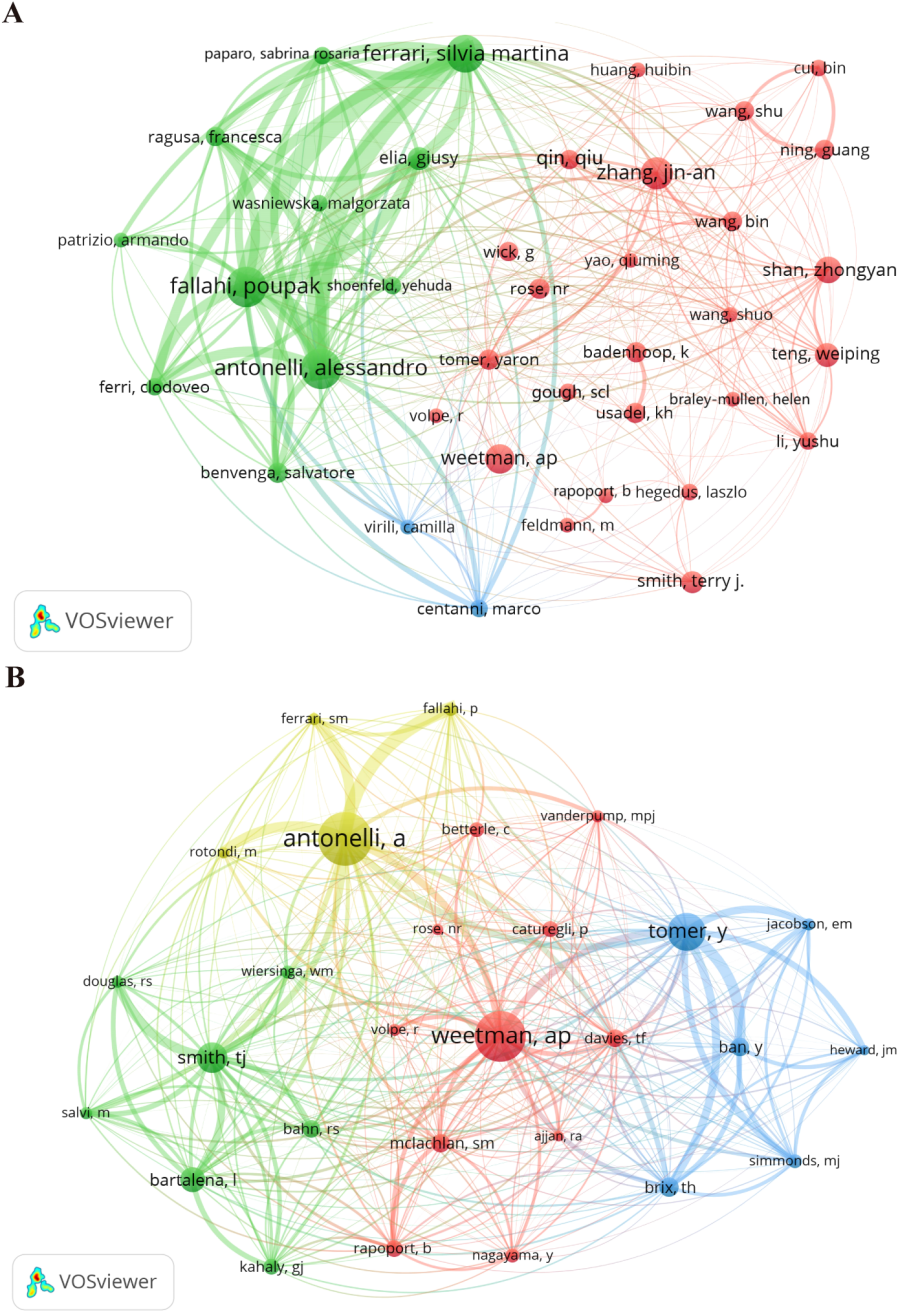
The visualization of authors **(A)** and co-cited authors **(B)** on research of AD and AIT. **(A)** a collaborative network was constructed based on 38 researchers whose number of published documents is more than or equal to 8 (attraction/repulsion values: 1/-3). Node size corresponds to the number of publications per author, different colors represent collaborative clusters or institutional affiliations, and line thickness indicates the strength of co-authorship relationships. **(B)** this study selected 28 authors to map the co-citation network based on minimum co-citations equals to 80 (attraction/repulsion values: 1/-3). Node size reflects total co-citation frequency, colors distinguish different research communities or temporal periods, and connecting lines show co-citation relationships with thickness representing co-citation strength.

The authorship landscape exhibited a conspicuous concentration of productivity within a collaborative research cluster, with Antonelli, Alessandro (30 articles, 2,382 citations, h-index=17), Fallahi, Poupak (30 articles, 2,366 citations, h-index=17), and Ferrari, Silvia Martina (27 articles, 2,227 citations, h-index=16) dominating the upper ranks through near-identical publication volumes and citation metrics, strongly suggestive of sustained collaborative partnerships, likely originating from the previously identified University of Pisa consortium (**Figure 5A**; **Table 3**). A pronounced heterogeneity in research impact emerged across the cohort, as exemplified by Zhang, Jin-An’s substantial output (23 articles) yielding disproportionately modest citation impact (380 citations, h-index=10)—approximately six-fold lower per-article citation than top-ranked authors—indicating potential variations in research novelty, methodological rigor, or target journal selectivity. The geographic composition reflected a Euro-Asian research axis, with established Western investigators (Weetman, Anthony P.; Smith, Terry J.; Badenhoop, K.) maintaining elevated h-indices relative to their publication counts, suggesting historical influence and foundational contributions, whereas emerging Asian scholars (Shan, Zhongyan; Teng, Weiping) demonstrated expanding productivity with comparatively nascent citation trajectories.

**Table 3:** Top 10 authors and co-cited authors on the research of AD and AIT.

Rank	Author	Counts	Citations	H-index	Co-cited authors	Citations
1	Antonelli, Alessandro	30	2382	17	Antonelli, A.	568
2	Fallahi, Poupak	30	2366	17	Weetman, A.P.	532
3	Ferrari, Silvia Martina	27	2227	16	Tomer, Y.	376
4	Zhang, Jin-An	23	380	10	Smith, T.J.	290
5	Weetman, Anthony P.	20	989	14	Bartalena, L.	233
6	Shan, Zhongyan	17	235	9	Brix, T.H.	169
7	Teng, Weiping	15	215	9	McLachlan, S.M.	159
8	Elia, Giusy	14	733	10	Ban, Y.	158
9	Smith, Terry J.	13	563	11	Davies, T.F.	151
10	Badenhoop, K	12	872	10	Bahn, R.S.	150

The co-citation analysis revealed a knowledge validation pattern wherein actively publishing scholars (Antonelli, A.: 568 co-citations; Weetman, A.P.: 532 co-citations) simultaneously occupied positions as frequently co-referenced authorities, indicating their dual role as contemporary contributors and established intellectual pillars within the AD-AIT research ecosystem (**Figure 5B**; **Table 3**). The prominence of pioneering figures such as Tomer, Y. (376 co-citations), Smith, T.J. (290 co-citations), and classical researchers including Bartalena, L. and Davies, T.F. underscores the field’s reliance on foundational mechanistic and clinical investigations, wherein current scholarship is systematically anchored to seminal works elucidating autoimmune pathogenesis, thyroid immunology, and disease classification frameworks.

### 3.5 Top co-cited references

**Figure 6:**
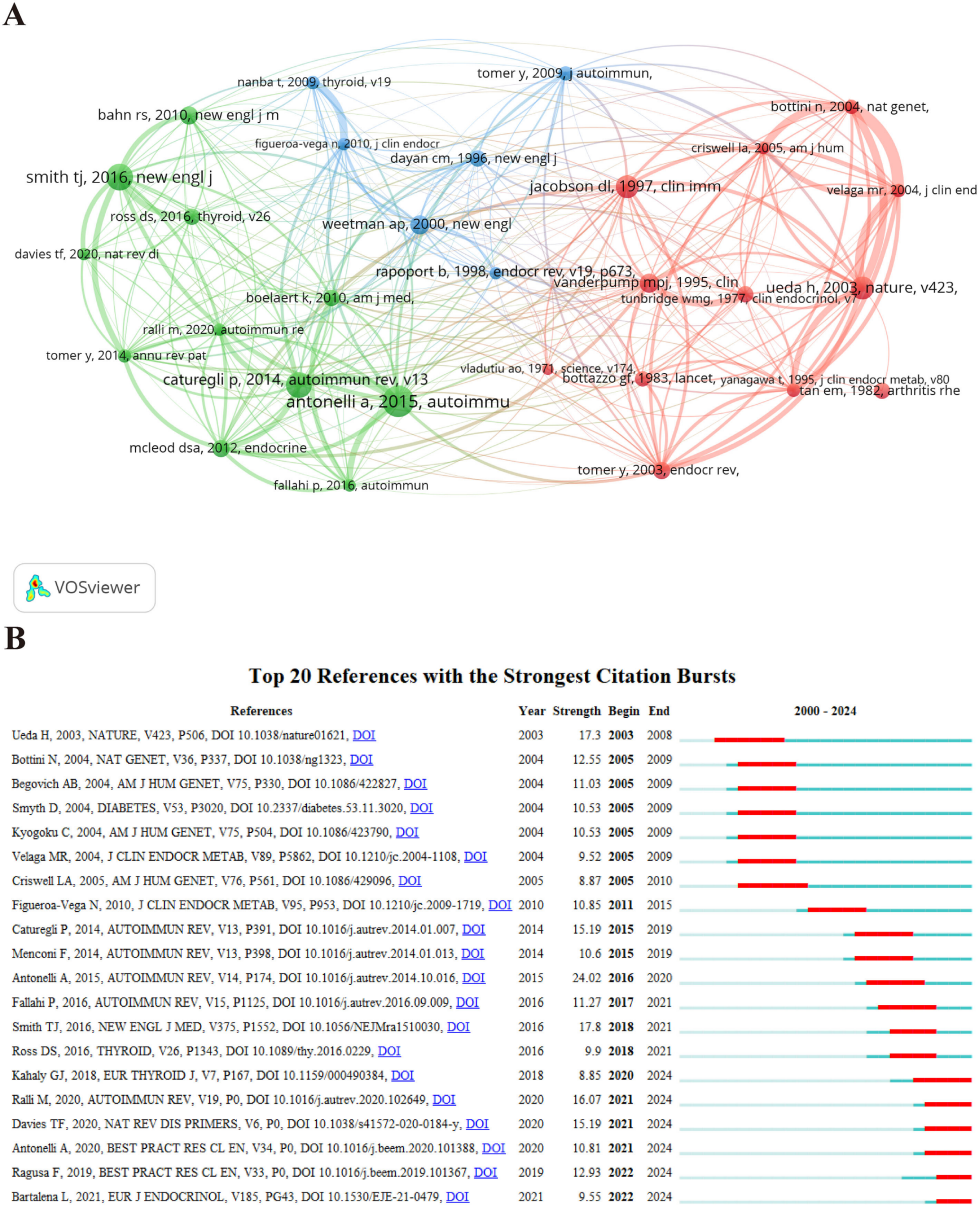
The visualization of co-cited references on research of AD and AIT. **(A)** A collaborative network was constructed based on 29 references whose number of co-citation is more than or equal to 35 (attraction/repulsion values: 2/-3). Node size represents total co-citation frequency, different colors indicate thematic clusters or research periods, and line thickness shows the strength of co-citation relationships between references. **(B)** Top 20 references with strong citation bursts [this study used the g-index (k=25) for node selection and applied a time slicing range from 2000 to 2024 (1-year per slice) to analyze the research trends]. A red bar indicates high citations in that year, with bar width reflecting citation intensity and blue bars representing baseline citation periods.

In terms of co-cited reference analysis, the article by Antonelli et al. (2015, *Autoimmun Rev*, v14, p174) has been cited 91 times (**Figure 6A**; **Supplementary Table 1**). Furthermore, in the “references with the strongest citation bursts” analysis, this article exhibits the highest citation burst strength (strength = 24.02) (**Figure 6B**). This paper focuses on the association between AITD and various systemic ADs as well as cancers, such as RA, SLE, SS, and papillary thyroid carcinoma (26). Moreover, strong collaboration is observed between “Velaga MR, 2004, *J Clin Endocr Metab*, v89, p5862” (27), “Bottini N, 2004, *Nat Genet*, v36, p337” (28), and “Ueda H, 2003, *Nature*, v423, p506” (29) (**Figure 6A**). These studies highlight the involvement of various immune-regulatory genes (such as *LYP*, *PTPN22*, and *CTLA4*) in ADs, which may play a role in the development of AIT and other ADs like Graves’ disease (GD) and T1D by influencing T cell function and immune tolerance mechanisms. The findings suggest a potential overlap in the genetic susceptibility and immunopathological processes of AIT and other ADs, especially in how shared genetic variants contribute to immune-mediated damage across different organs. This opens up a new avenue for research into the systemic factors and early prediction of AIT.

### 3.6 Current hotspots and key topics on AD-AIT fields

**Figure 7:**
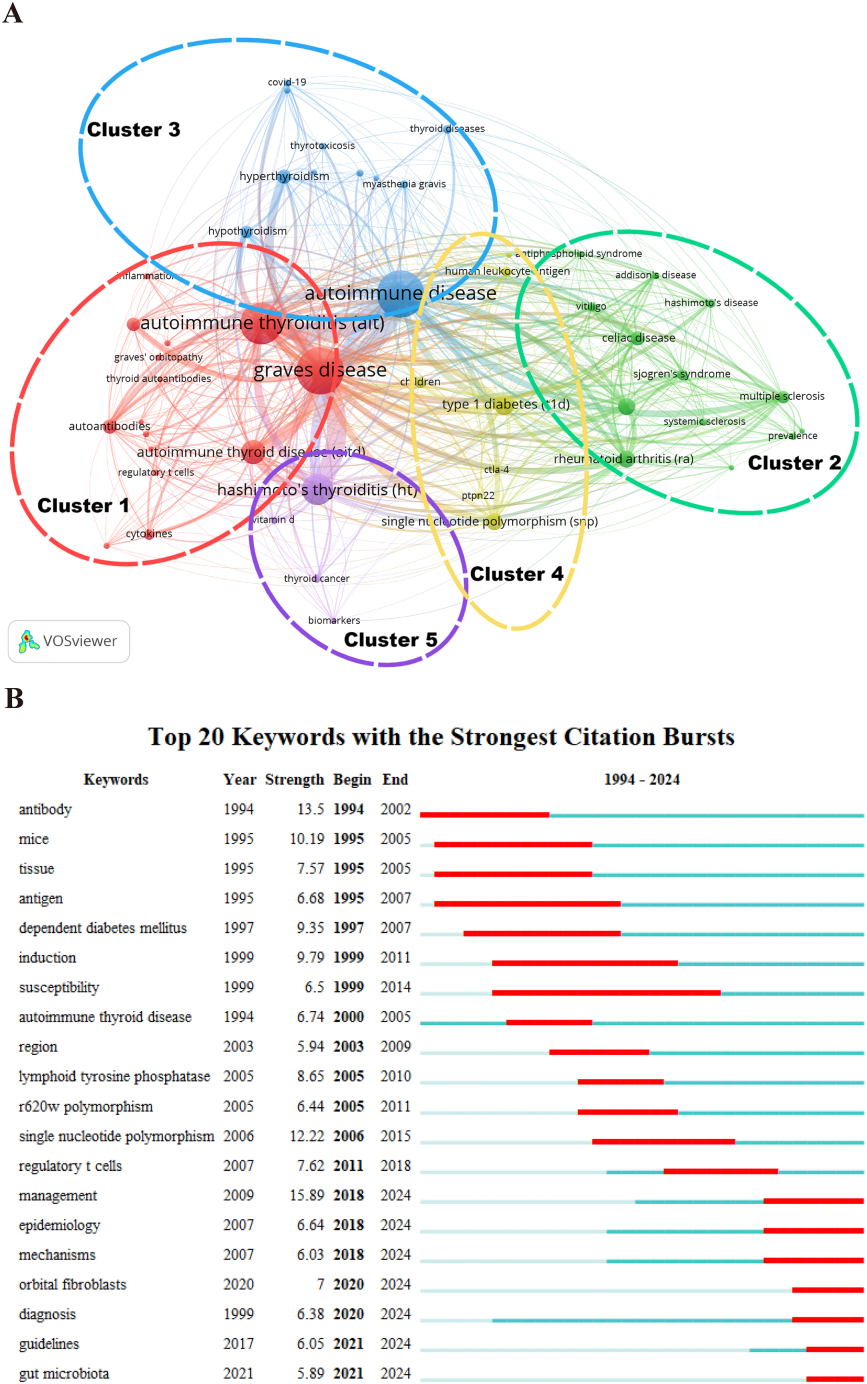
Keywords visualization analysis using VOSviewer. **(A)** Keyword cluster analysis [more than 13 occurrences (50 keywords) were filtered to map the network (attraction/repulsion values = 0/-1)]. Node size reflects keyword frequency, different colors represent distinct research theme clusters, line thickness indicates co-occurrence strength between keywords, and spatial proximity shows thematic similarity. **(B)** Top 20 keywords with strongest citation bursts [this study used the g-index (k=25) for node selection and applied a time slicing range from 1994 to 2024 (1-year per slice) to analyze the research trends]. Red bars indicate periods of increased citation activity (bursts), blue bars show baseline periods, and bar thickness represents burst strength intensity.

**Figure 8:**
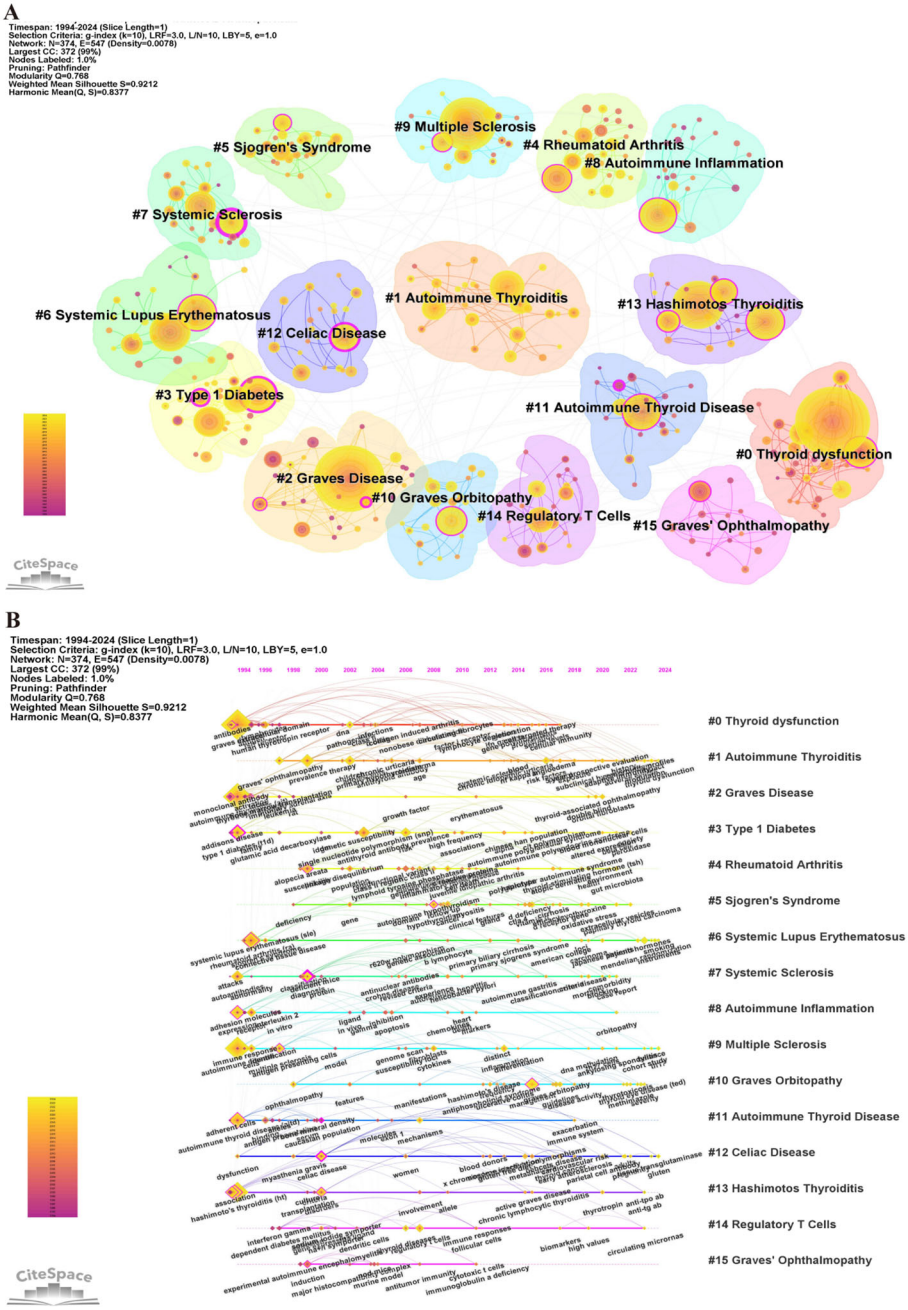
Keywords analysis related to AD and AIT fields. **(A)** Cluster analysis of keywords, visualized with CiteSpace. The network consists of 374 nodes and 547 edges, with a modularity Q score of 0.768 and a weighted mean silhouette score of 0.9212, indicating good clustering quality. **(B)** Timeline view.

Keyword analysis identified and visualized five main research themes (**Figure 7A**; **Supplementary Table 2**), including immune dysregulation and inflammatory mechanisms, comorbidities and shared pathological mechanisms, immune interference due to infections and pregnancy, genetic susceptibility and mechanisms, and biomarkers and cancer risks. The timeline view, cluster analyses and trend topics of keywords further emphasize the ongoing focus on the relationship between major ADs and AIT, along with their clinical characteristics and treatment (**Figures 8A, B**; **Supplementary Figure 2**). Citation-burst analysis from 2018 to 2024 identifies that future research should continue to prioritize disease management, pathogenesis, diagnosis, and treatment guidelines, offering valuable direction for studies in this area (**Figure 7B**).

Furthermore, the thematic map shows the evolution and relevance of key topics in this field (**Supplementary Figure 3**). The lower-right quadrant highlights areas that remain underexplored, suggesting that future studies should focus on immune regulation and inflammation mechanisms, the role of gut microbiota in immune regulation, genetic susceptibility and gene polymorphisms, the relationship between comorbidities and systemic ADs, and biomarkers with causal inference.

## 4 Discussion

### 4.1 General information

This study provides the first comprehensive bibliometric analysis of AD and AIT research, a field that has historically lacked systematic focus. Our findings highlight the rapid growth and increasing interest in this area, particularly with a significant surge in research activity since 2016. This growth reflects the substantial support and strategic commitment from leading global medical research institutions.

The analysis identifies distinct geographic patterns in publication volume and research impact. The United States leads in total output, followed by China and Italy. While China currently has a lower citation rate than some Western nations, its consistent high publication volume and steadily increasing citation impact (strength = 28.08) (**Supplementary Figure 4**) demonstrate its growing influence. Prominent Chinese institutions, such as Fudan University (strength = 9.04) (**Supplementary Figure 5**), and leading researchers (**Supplementary Figure 6**) further underscore China’s significant role in advancing this field. Enhancing international collaborations and focusing on high-quality research will help China bridge the existing citation gap.

Regarding journal performance, the *Journal of Clinical Endocrinology & Metabolism* leads in publication volume, notwithstanding its moderate impact factor (IF = 5.1). As the most frequently co-cited journal, it illustrates the strong link between sustained publication volume and long-term academic influence. The high citation performance of premier journals—such as the *Lancet*, *New England Journal of Medicine*, and *Nature*—highlights their essential role in disseminating AD and AIT research. Therefore, researchers seeking to maximize their impact should prioritize publishing in these influential venues over simply increasing their total publication count.

### 4.2 Emerging trends and future directions

#### 4.2.1 Current research hotspots and knowledge structure

**Figure 9:**
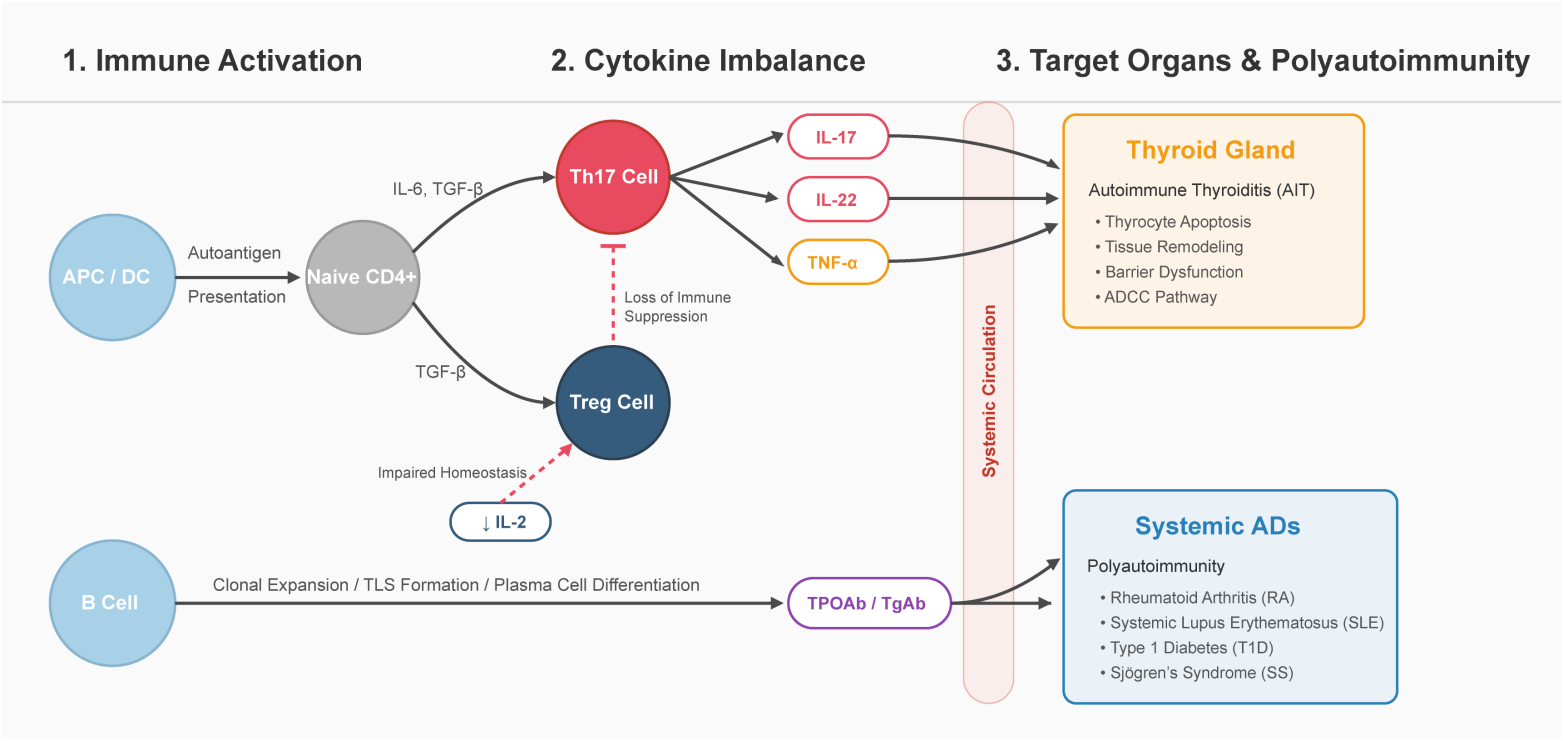
The interconnected cytokine and humoral immune network in AIT and systemic polyautoimmunity. The schematic illustrates how Th17/Treg imbalance (exacerbated by IL-2 deficiency) and elevated pro-inflammatory cytokines (IL-6, TNF-α, IL-17, and IL-22) coordinate thyroid follicular destruction. Concurrently, autoreactive B cells undergo clonal expansion and form ectopic tertiary lymphoid structures (TLS), generating high-titer autoantibodies (TPOAb/TgAb). These pathogenic cellular and humoral components enter the systemic circulation to drive localized autoimmune thyroiditis (AIT) and multi-organ polyautoimmunity (RA, SLE, T1D, and SS).

Keyword clustering analysis identified five interconnected thematic domains that illustrate the complex relationship between AD and AIT (**Figure 7A**). Cluster 1 emphasizes the shared pathological foundation of immune dysregulation and inflammation. In this process, the destruction of apoptotic thyrocytes releases thyroid-specific antigens, triggering the production of autoantibodies—a hallmark feature common to multiple AD entities (30). Cytokine imbalances, especially elevated pro-inflammatory mediators (*IL-6*, *TNF-α*) and impaired Treg function, serve as critical pathomechanistic links between AIT and systemic autoimmunity (31). Crucially, while T-cell-mediated cytotoxicity and Treg dysfunction are central drivers of cellular destruction, B-cell-mediated humoral immunity represents a fundamental component in the pathogenesis of AIT. A characteristic clinical hallmark of AIT is the high-titer production of circulating polyclonal autoantibodies against thyroid peroxidase (TPOAb) and thyroglobulin (TgAb) (32–35). Evidence indicates that under chronic inflammation, autoreactive B cells clonally expand in non-lymphoid tissues, forming ectopic tertiary lymphoid structures (TLS). Resembling secondary lymphoid organs, TLS contain B, T, and dendritic cell zones that sustain local adaptive immunity and drive persistent tissue inflammation (36–39). Beyond their classical terminal differentiation into antibody-secreting plasma cells, B cells function as antigen-presenting cells (APCs). They internalize thyroid self-antigens via clonal B-cell receptors (BCRs) and provide costimulatory signals to naive CD4+ T cells, which may sustain localized immune activation (40–44). Furthermore, these autoantibodies can initiate antibody-dependent cell-mediated cytotoxicity (ADCC) and complement activation, contributing to the apoptosis of thyroid follicular epithelial cells (45–47). In polyautoimmunity, dysregulation of the *BAFF/APRIL* system—essential survival factors for peripheral B cells—serves as a shared molecular axis linking AIT with systemic ADs like SLE and RA, highlighting B-cell pathways as potential therapeutic targets (48–52). Expanding this cytokine landscape, emerging evidence highlights the pathogenic contributions of additional interleukins: *IL-2* deficiency or signaling dysregulation impairs Treg homeostasis and peripheral immune tolerance, potentially fostering autoantigen-specific T cell expansion in AIT (53–55); The Th17/Treg imbalance serves as a critical prognostic factor for thyroid glandular damage, functioning as a primary determinant of persistent inflammation and defective immune suppression. Within this pathological framework, Th17 cells—the predominant source of *IL-17*—potentiate the progression of HT by amplifying local cytokine cascades and escalating the inflammatory response. Elevated levels of *IL-17* further exacerbate glandular injury, reflecting a state where the pro-inflammatory Th17 axis overwhelms the counter-regulatory mechanisms of Treg cells (55, 56); and *IL-22*, acting at epithelial interfaces, may contribute to thyroid tissue remodeling and barrier dysfunction, further sustaining chronic autoimmune activation (57). Evaluating the levels and functional roles of *IL-2*, *IL-17*, and *IL-22* in prospective AIT cohorts represents a clinically meaningful research priority, as these cytokines are already therapeutic targets in related autoimmune conditions and may offer novel intervention points in AIT-associated polyautoimmunity. The intricate interplay within this cytokine-humoral network and its systemic pathological relevance are comprehensively integrated in **Figure 9**. Accumulating evidence implicates gut microbiota dysbiosis and intestinal inflammation as potential mechanistic bridges connecting AIT with T1D and RA, wherein microbial metabolites and bacterial translocation may precipitate systemic immune activation (58–61).

Cluster 2 highlights the significant comorbidity burden between AIT and various ADs, suggesting convergent genetic and immunopathological mechanisms. HT frequently coexists with SLE, RA, and SS, pointing to shared immune regulatory dysfunction as a unifying factor (62). The phenomenon of polyautoimmunity—particularly the concurrence of vitiligo and AIT—exemplifies a systemic autoimmune predisposition (63). Celiac disease also shows HLA gene associations that parallel those in AIT, leading to cross-reactive immune dysregulation (64, 65). Notably, comorbidity patterns vary by age: connective tissue disorders are more common in adult-onset AIT (10), while T1D and celiac disease are more prevalent in pediatric and adolescent populations (66, 67).

Cluster 3 investigates the immunological links and mechanisms connecting AIT with related ADs. Thyroid autoantibodies are essential diagnostic and prognostic markers that can disrupt thyroid homeostasis, leading to thyrotoxicosis or hypothyroidism (68). Pregnancy also introduces unique challenges, as maternal AIT increases the risk of gestational complications for both mother and fetus (69). Recent studies suggest that SARS-CoV-2 infection may trigger or worsen AIT through excessive immune activation or direct thyroidal injury (70). Furthermore, the presence of conditions like myasthenia gravis in AIT patients underscores immune dysregulation that extends beyond a single organ (71).

Cluster 4 delineates shared genetic susceptibility mechanisms. Single nucleotide polymorphisms (SNPs) in *CTLA-4* and *PTPN22* genes are robustly associated with T1D, AIT, and related disorders, making these loci as pleiotropic susceptibility factors across the AD spectrum (61, 72). HLA system polymorphisms are pervasive in AIT and T1D, supporting the idea that immune tolerance breakdown is a systemic phenomenon (73). Pediatric studies reveal elevated AIT-T1D comorbidity rates, potentially reflecting shared genetic foundations and convergent immune perturbations during developmental windows (74). Mendelian randomization analyses now provide causal evidence for bidirectional relationships between AIT and several systemic ADs, with *TYK2* gene variants emerging as pivotal therapeutic targets involved in Th17, Th1, and Th2 cell differentiation (75–77).

Cluster 5 examines the biomarker landscape in AIT pathophysiology. Beyond traditional antibodies, Vitamin D deficiency is highly prevalent in HT and related ADs, potentially modulating pathogenesis through immunoregulatory effects (78, 79). The gut microbiota-vitamin D axis represents a promising therapeutic frontier for attenuating inflammatory cascades (80). Notably, AIT patients face an increased risk of thyroid malignancies, particularly papillary thyroid carcinoma, requiring vigilant surveillance (81). Finally, multi-omics investigations reveal complex metabolic derangements in HT, including disrupted tryptophan and tyrosine metabolism, as well as lipid dysregulation affecting sphingomyelin and phosphatidylcholine profiles (82–84).

These research domains are intricately linked. For instance, AIT comorbidity with SLE and RA likely stems from Treg dysfunction and cytokine overproduction. Infectious agents may trigger AIT through epigenetic modifications (DNA methylation) that affect susceptibility genes like *CTLA-4* and *PTPN22*. Pregnancy-associated immune fluctuations, combined with genetic and infectious factors, may further amplify AIT and broader autoimmune risks, although causal directionality requires confirmation through longitudinal mechanistic studies. The observed bibliometric co-clustering of thyroid autoantibody research with oncology topics suggests investigative interest in the association between chronic thyroid inflammation and malignancy risk; however, it should be emphasized that bibliometric co-citation patterns reflect shared research attention rather than established causation, and mechanistic claims regarding carcinogenesis remain to be validated through experimental and clinical evidence.

#### 4.2.2 Emerging research trends

##### 4.2.2.1 Precision medicine and multi-omics integration

Precision medicine is transforming AD-AIT management. Beyond traditional levothyroxine therapy, new evidence supports the use of myoinositol and selenium to reduce TSH and autoantibody levels (85, 86). Artificial intelligence (AI)-enhanced diagnostics also show promise in identifying antibody-negative AIT, helping to overcome challenges in seronegative cases (87). Multi-omics platforms—integrating genomics, transcriptomics, proteomics, and metabolomics—allow for more precise disease phenotyping and patient stratification. Machine learning models can use this high-throughput data to personalize levothyroxine doses and predict treatment responses. Furthermore, metabolomic profiling has identified disruptions in bile acid, tryptophan, and lipid metabolism, offering potential new therapeutic targets.

##### 4.2.2.2 Microbiome-immune axis and post-infectious autoimmunity

**Figure 10:**
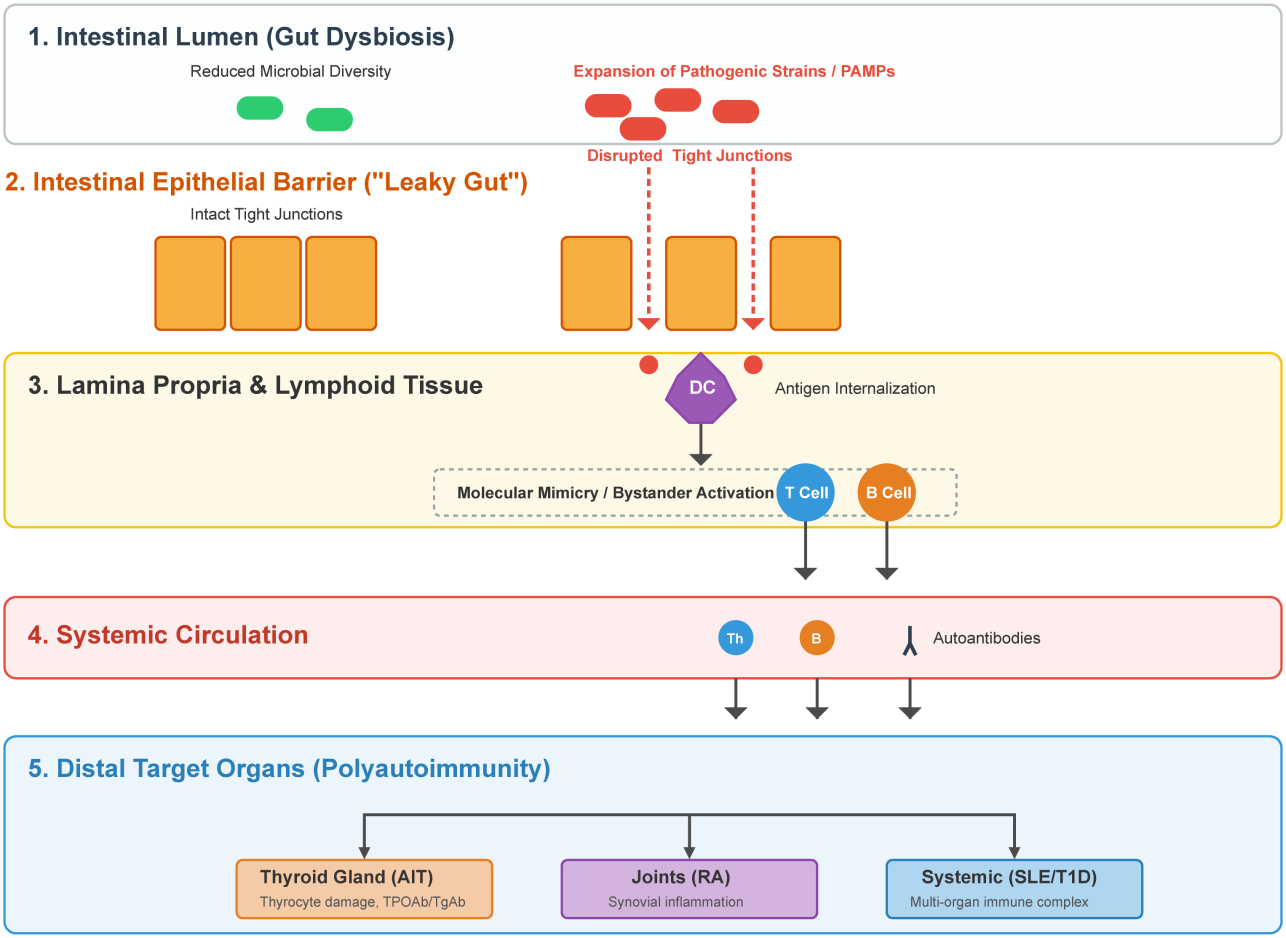
Proposed pathological mechanisms of the gut-thyroid axis in polyautoimmunity. Gut dysbiosis and reduced microbial diversity disrupt epithelial tight junctions, inducing a “leaky gut” state that facilitates the translocation of microbial antigens into the lamina propria. Translocated antigens are internalized by dendritic cells (DCs), triggering the activation and clonal expansion of autoreactive T and B cells via molecular mimicry or bystander activation. These pathogenic lymphocytes and autoantibodies disseminate through the systemic circulation, driving both localized AIT and remote systemic autoimmune comorbidities.

Gut microbiota dysbiosis is a key factor influencing vitamin D metabolism, intestinal permeability, and systemic inflammation. Molecular mimicry between gut and thyroid antigens—especially in celiac-related thyroiditis—suggests that microbiome modulation could be an innovative treatment strategy (88). Moving beyond conceptual frameworks, recent evidence highlights the therapeutic potential of directly targeting this axis. Modulating the gut microbiota through targeted probiotics or structured dietary shifts has been shown to support the maintenance of intestinal epithelial barrier integrity, potentially limiting the translocation of microbial antigens into systemic circulation (89–91). By modulating the mucosal barrier, these microbiome-based interventions may influence peripheral dendritic cell activation and help attenuate systemic inflammatory pathways (92, 93). These microecological strategies and tailored nutritional approaches represent an emerging translational area of interest for managing the gut-thyroid axis and its associated polyautoimmune presentations. The proposed multi-layer anatomical and immunological cascade spanning from the gut lumen to distal target organs is detailed in **Figure 10**. Experience with COVID-19 has highlighted how infections can trigger autoimmunity; cases of new-onset AIT and GD have been reported following SARS-CoV-2 infection due to molecular mimicry and immune hyperactivation (94). These findings suggest that infectious triggers are modifiable risk factors that require closer surveillance and further study.

#### 4.2.3 Future research directions

Priority areas include: (1) investigating the mechanisms of polyautoimmunity using single-cell multi-omics and longitudinal cohorts to understand how AD-AIT clusters form; (2) developing biomarker panels that integrate genetic risk scores (*HLA*, *CTLA-4*, *PTPN22*, *TYK2*), cytokine profiles, and metabolic signatures for personalized risk assessment; (3) creating new therapies that target Treg dysfunction and cytokine pathways (such as anti-*TNF* and *TYK2-JAK-STAT*) based on specific disease profiles (95–97); (4) identifying at-risk individuals through genetic screening and autoantibody monitoring in relatives to test if early intervention can prevent clinical disease; and (5) studying the brain-thyroid axis using neuroimaging and omics to understand the causes of neuropsychiatric symptoms.

#### 4.2.4 Challenges and solutions

Key challenges include: (1) complex disease variations that make diagnosis difficult, which may be addressed using machine learning for subtyping and standardized international registries; (2) the slow transition of biomarker discoveries into clinical use, requiring rigorous validation and standardized testing platforms; (3) barriers to integrating multi-omics data, which require cloud-based platforms and standardized data-sharing; (4) an incomplete understanding of polyautoimmunity, requiring long-term studies and deep immune profiling to identify early causes; and (5) gaps in implementing personalized treatments, which can be bridged through biomarker-driven trials and clinical support tools.

In summary, advancing AD-AIT research requires integrating precision diagnostics with targeted therapies while overcoming translational hurdles through multidisciplinary collaboration focused on patient outcomes.

### 4.3 Limitations and future directions

#### 4.3.1 Research limitations

This bibliometric investigation, while comprehensive, exhibits several inherent constraints. First, database selection encompassed PubMed, WoSCC, and Scopus, potentially omitting relevant publications indexed exclusively in regional or specialized databases (Embase, CNKI, SciELO), thereby introducing geographic and linguistic representation biases. Second, the analysis predominantly captured English-language publications, potentially underrepresenting significant contributions from non-English scholarly communities, particularly those addressing region-specific AD-AIT epidemiology and genetic susceptibility patterns. Third, citation metrics exhibit temporal lag effects, wherein recently published high-quality studies (2022-2024) demonstrate artificially deflated citation counts relative to their scientific merit, potentially obscuring emerging research frontiers. Fourth, bibliometric methodologies prioritize quantitative publication metrics over qualitative research rigor, innovation, or clinical translatability, potentially overvaluing prolific but methodologically modest contributions while undervaluing transformative but selectively published investigations. Fifth, keyword-based analyses depend on author-assigned terminologies, which may lack standardization across disciplines, institutions, and temporal periods, potentially fragmenting thematically related research clusters; consequently, the five identified thematic domains should be interpreted as reflecting patterns in keyword co-occurrence rather than as definitive scientific taxonomies, since terminology drift across decades may introduce classification artifacts. Sixth, the data included in the analysis, which terminated on December 31, 2024, provide a static snapshot of a dynamically evolving field, underscoring the need for periodic updates to maintain contemporary relevance. Seventh, the analytical period extends to 1980, a time when AIT diagnostic criteria and disease nomenclature were considerably less standardized than today; publications from the 1980–2000 foundational phase should therefore be interpreted cautiously, as terminological heterogeneity may limit direct comparability with modern literature. Eighth, the present analysis does not include a comparative cohort from other autoimmune or endocrine conditions (e.g., GD, SLE in isolation, or T1D bibliometric profiles); the absence of such a reference benchmark means that trends observed here reflect the absolute trajectory of AIT-AD research without contextualizing whether these patterns are distinctive to this field or common across related autoimmune disciplines. Future analyses incorporating comparative bibliometric benchmarking would strengthen the field-specific conclusions.

#### 4.3.2 Future improvement directions

To address identified limitations and enhance analytical comprehensiveness, future bibliometric investigations should consider: (1) Expanded database integration incorporating multidisciplinary repositories (Embase, CINAHL, PsycINFO) alongside regional databases (CNKI for Chinese literature, J-STAGE for Japanese publications) to enhance global representation; (2) Multilingual inclusion strategies employing automated translation algorithms and native-speaker validation to incorporate non-English scholarship, particularly from emerging research economies; (3) Longitudinal monitoring frameworks implementing annual or biennial updates to track real-time research evolution, emerging collaborations, and paradigm shifts; (4) Hybrid methodological approaches integrating quantitative bibliometrics with qualitative systematic reviews, expert consensus panels, and content analyses to evaluate research quality, methodological rigor, and clinical applicability beyond citation metrics alone; (5) Subfield-specific analyses conducting focused investigations on discrete topics (genetic susceptibility, biomarker development, therapeutic interventions, pregnancy-related complications) to provide granular insights exceeding broad-spectrum overviews; (6) Altmetric integration incorporating social media engagement, policy citations, and clinical guideline references to assess societal impact and knowledge translation effectiveness beyond traditional academic metrics; (7) Predictive modeling employing machine learning algorithms to forecast emerging research themes, identify underexplored domains with high future potential, and project collaborative network evolution to guide strategic research investment.

In summary, this comprehensive bibliometric analysis systematically mapped the global research landscape of AD and AIT across 1980-2024, encompassing 2,201 publications from 95 countries. The investigation revealed exponential growth post-2016, driven by enhanced international collaboration and multi-omics technology advancement. Geographic analysis demonstrated United States leadership in publication volume and citation impact, while Western European institutions exhibited superior per-article citation efficiency.

Thematic analyses identified five interconnected research domains: immune dysregulation and inflammatory mechanisms, comorbidity patterns with T1D/RA/SLE/SS, pregnancy and infection-triggered autoimmunity (including SARS-CoV-2), genetic susceptibility mechanisms (*CTLA-4*, *PTPN22*, *HLA*, *TYK2*), and biomarker landscapes encompassing metabolomic signatures and malignancy risk. Emerging trends emphasize precision medicine integrating multi-omics platforms, AI-enhanced diagnostics, and personalized therapeutic algorithms.

Future research priorities include mechanistic polyautoimmunity elucidation through single-cell technologies and Mendelian randomization, composite biomarker panel development, novel immunomodulatory therapies targeting Treg dysfunction, preventive strategies for at-risk populations, and brain-thyroid axis investigations. Critical challenges encompass phenotypic heterogeneity, limited biomarker translation, and personalized treatment implementation gaps, necessitating collaborative multidisciplinary solutions. These findings provide evidence-based guidance for strategic research optimization, ultimately advancing comprehensive understanding of AIT’s role within the AD spectrum and informing more effective clinical management strategies for patients with polyautoimmunity.

## Data Availability

The original contributions presented in the study are included in the article/**Supplementary Material**. Further inquiries can be directed to the corresponding author.
