# Supplementary material for: Autoimmune thyroiditis and comorbid autoimmune diseases: global research landscape and future directions
Source: Front Immunol. 2026 Jul 16;17:1746955. doi: 10.3389/fimmu.2026.1746955 (PMC13422399; doi:10.3389/fimmu.2026.1746955)
Supplement: Supplementary file 1 [file DataSheet1.pdf]

Scopus (n=4,102): (Title-Abstract-Keywords ("Autoimmune Disease*" OR "Connective Tissue Disease" OR "Mixed Connective Tissue Disease" OR "Systemic Sclerosis" OR "Scleroderma" OR "Rheumatoid Arthritis" OR "Sjogren* Syndrome" OR "Polymyositis" OR "Dermatomyositis" OR "Myositis*" OR "Systemic Lupus Erythematosus" OR "Antiphospholipid Syndrome") AND Title-Abstract-Author Keywords ("Autoimmune Thyroiditides" OR "Thyroiditides, Autoimmune" OR "Thyroiditis, Lymphocytic" OR "Lymphocytic Thyroiditides" OR "Lymphocytic Thyroiditis" OR "Thyroiditides, Lymphocytic" OR "Autoimmune Thyroiditis" OR "Hashimoto* Thyroiditis" OR "Graves* Disease")) AND (PUBYEAR (1980-2024))

Pubmed (n=3,326): (Title-Abstract ("Autoimmune Disease*" OR "Connective Tissue Disease" OR "Mixed Connective Tissue Disease" OR "Systemic Sclerosis" OR "Scleroderma" OR "Rheumatoid Arthritis" OR "Sjogren* Syndrome" OR "Polymyositis" OR "Dermatomyositis" OR "Myositis*" OR "Systemic Lupus Erythematosus" OR "Antiphospholipid Syndrome") AND (Title-Abstract ("Autoimmune Thyroiditides" OR "Thyroiditides, Autoimmune" OR "Thyroiditis, Lymphocytic" OR "Lymphocytic Thyroiditides" OR "Lymphocytic Thyroiditis" OR "Thyroiditides, Lymphocytic" OR "Autoimmune Thyroiditis" OR "Hashimoto* Thyroiditis" OR "Graves* Disease")) AND (PUBYEAR (1980-2024))

WoSCC (n=3,565): (Title-Abstract-Author Keywords ("Autoimmune Disease*" OR "Connective Tissue Disease" OR "Mixed Connective Tissue Disease" OR "Systemic Sclerosis" OR "Scleroderma" OR "Rheumatoid Arthritis" OR "Sjogren* Syndrome" OR "Polymyositis" OR "Dermatomyositis" OR "Myositis*" OR "Systemic Lupus Erythematosus" OR "Antiphospholipid Syndrome") AND Title-Abstract-Author Keywords ("Autoimmune Thyroiditides" OR "Thyroiditides, Autoimmune" OR "Thyroiditis, Lymphocytic" OR "Lymphocytic Thyroiditides" OR "Lymphocytic Thyroiditis" OR "Thyroiditides, Lymphocytic" OR "Autoimmune Thyroiditis" OR "Hashimoto* Thyroiditis" OR "Graves* Disease")) AND (PUBYEAR (1980-2024))

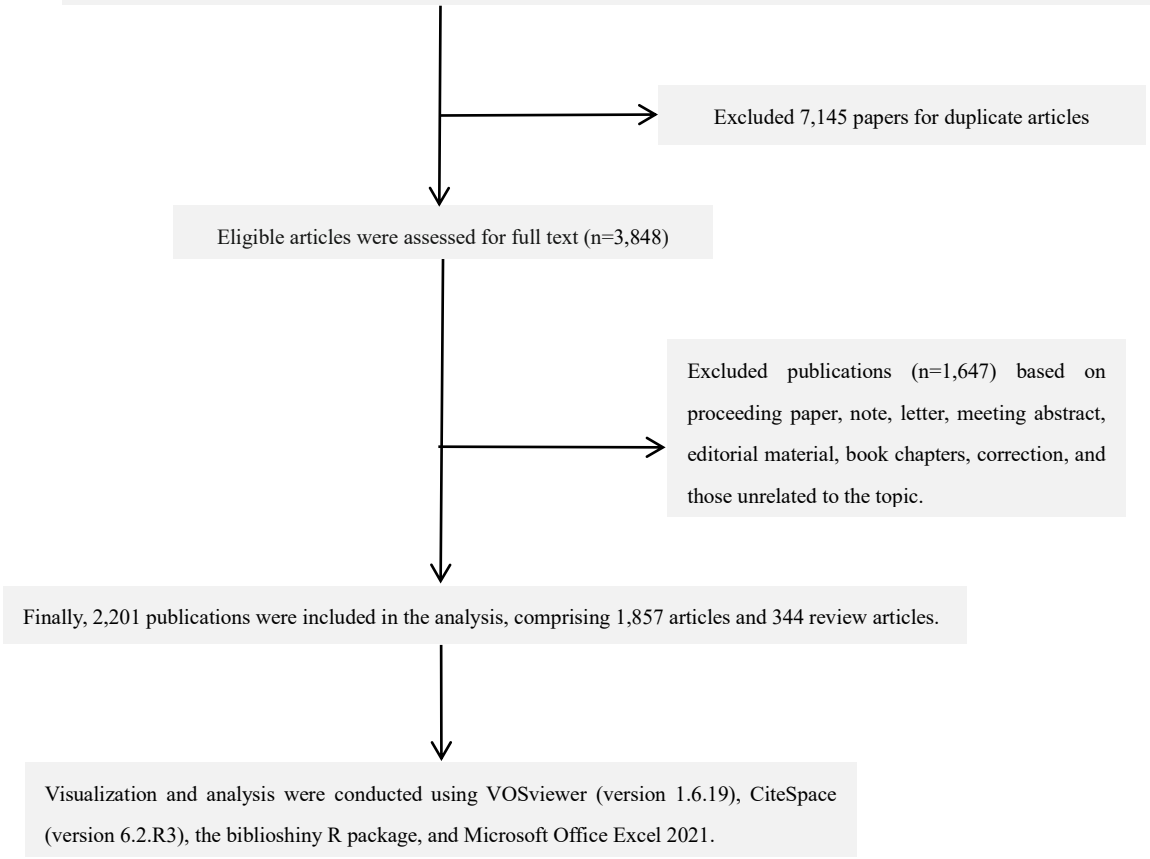

Supplementary Figure 1: Flow diagram of search strategy and study selection.

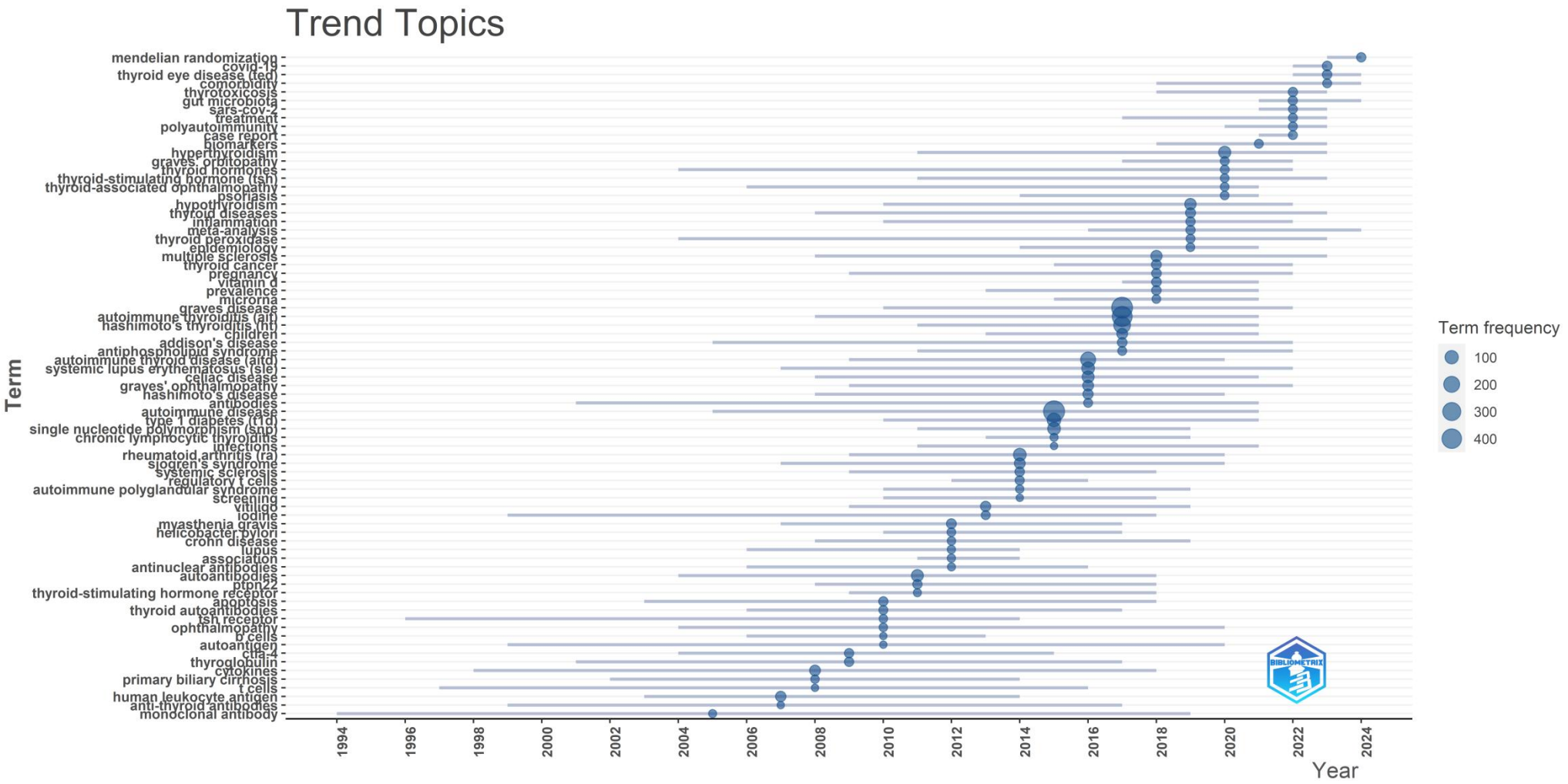

Supplementary Figure 2: Trends topics in AD and AIT research.

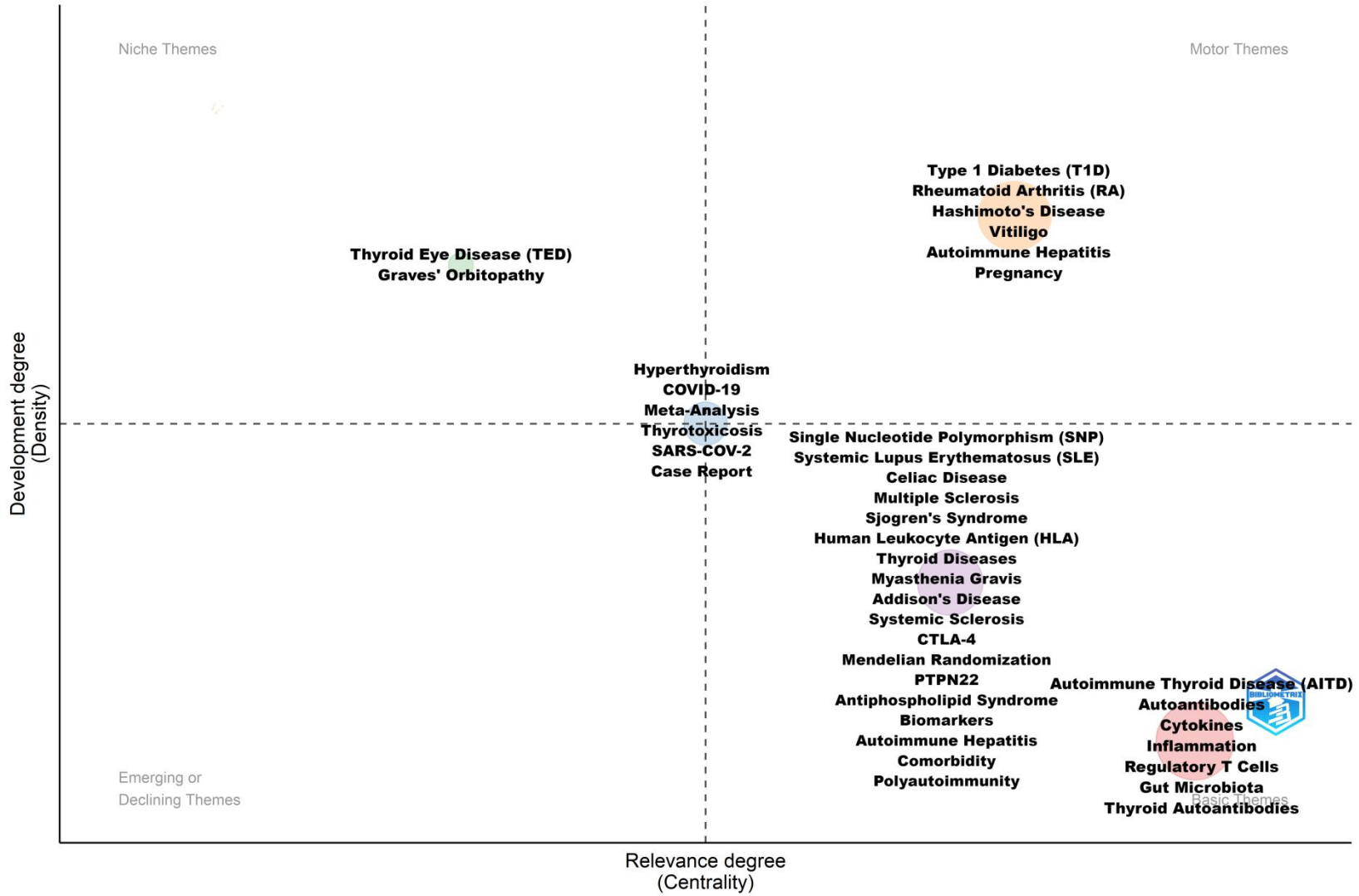

Supplementary Figure 3: Thematic map: The horizontal axis represents mediating centrality, indicating the theme's relevance to the field, while the vertical axis represents density, showing how well the theme has developed. Quadrant 1 (top right) represents MOTOR themes, which are both important and well-developed. Quadrant 2 (top left) represents HIGHLY DEVELOPED and INDIVIDUAL themes—those that are well-developed but less relevant to the current domain. Quadrant 3 (bottom left) includes EMERGING OR DECLINING themes, which are underdeveloped and may be either gaining or losing relevance. Quadrant 4 (bottom right) represents BASIC and TRANSVERSAL themes, which are important to the field but not yet well-developed.

Top 10 Countries with the Strongest Citation Bursts

Countries	Year	Strength	Begin	End	1994 - 2024
JAPAN	1994	6.78	**1994**	2004	
AUSTRIA	1995	4.93	**1995**	2003	
SPAIN	1996	4.74	**1996**	1998	
ENGLAND	1994	10.34	**1997**	2007	
TURKEY	1999	4.52	**2004**	2013	
GERMANY	1995	5.31	**2008**	2010	
ITALY	1994	6.85	**2017**	2018	
RUSSIA	1999	4.89	**2017**	2021	
PEOPLES R CHINA	1998	28.08	**2021**	2024	
INDIA	2005	8.51	**2021**	2024	

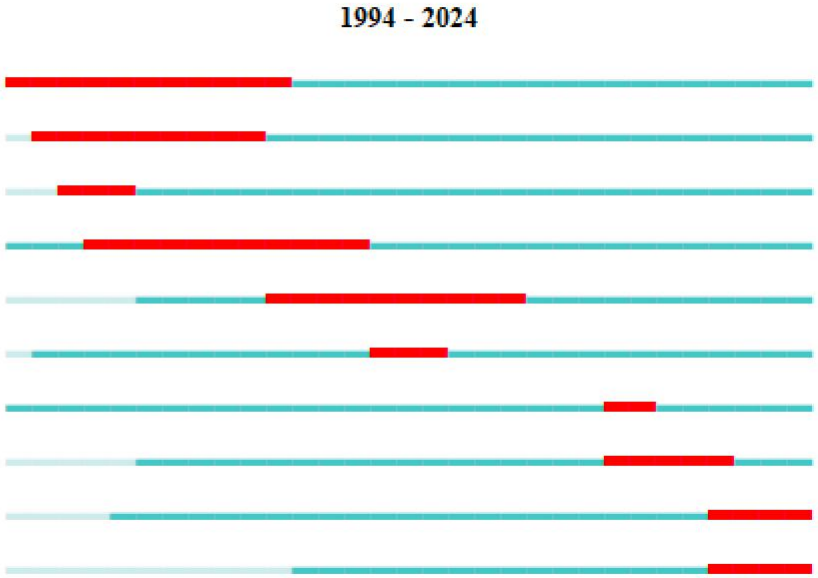

Supplementary Figure 4: Top 10 countries with strong citation bursts. A red bar indicates high citations in that year.

Top 20 Institutions with the Strongest Citation Bursts

Institutions	Year	Strength	Begin	End	1994 - 2024
Johns Hopkins University	1996	4.68	**1996**	2000	
University of Cambridge	1999	4.92	**1999**	2007	
University of Birmingham	1998	8.32	**2003**	2010	
University of Copenhagen	2000	4.58	**2006**	2013	
Veterans Health Administration (VHA)	2007	5.98	**2007**	2012	
US Department of Veterans Affairs	1996	5.78	**2007**	2012	
Chinese Academy of Sciences	2003	5.09	**2008**	2014	
Shanghai Jiao Tong University	2009	4.94	**2009**	2015	
University of Michigan	2009	4.74	**2009**	2015	
University of Michigan System	2009	4.74	**2009**	2015	
Medical University of Warsaw	2011	5.5	**2011**	2016	
Fudan University	2003	9.04	**2014**	2019	
Universita di Modena e Reggio Emilia	2008	5.42	**2014**	2017	
University of Messina	2015	6.19	**2015**	2024	
University of Pisa	2001	6.11	**2016**	2024	
Sapienza University Rome	1996	5.9	**2017**	2024	
China Medical University	2014	5.17	**2017**	2022	
Azienda Ospedaliero Universitaria Pisana	2014	5.16	**2017**	2024	
Shanghai University of Medicine & Health Sciences	2019	7.75	**2019**	2022	
Fujian Medical University	2022	4.85	**2022**	2024	

Supplementary Figure 5: Top 20 institutions with strong citation bursts. A red bar indicates high citations in that year.

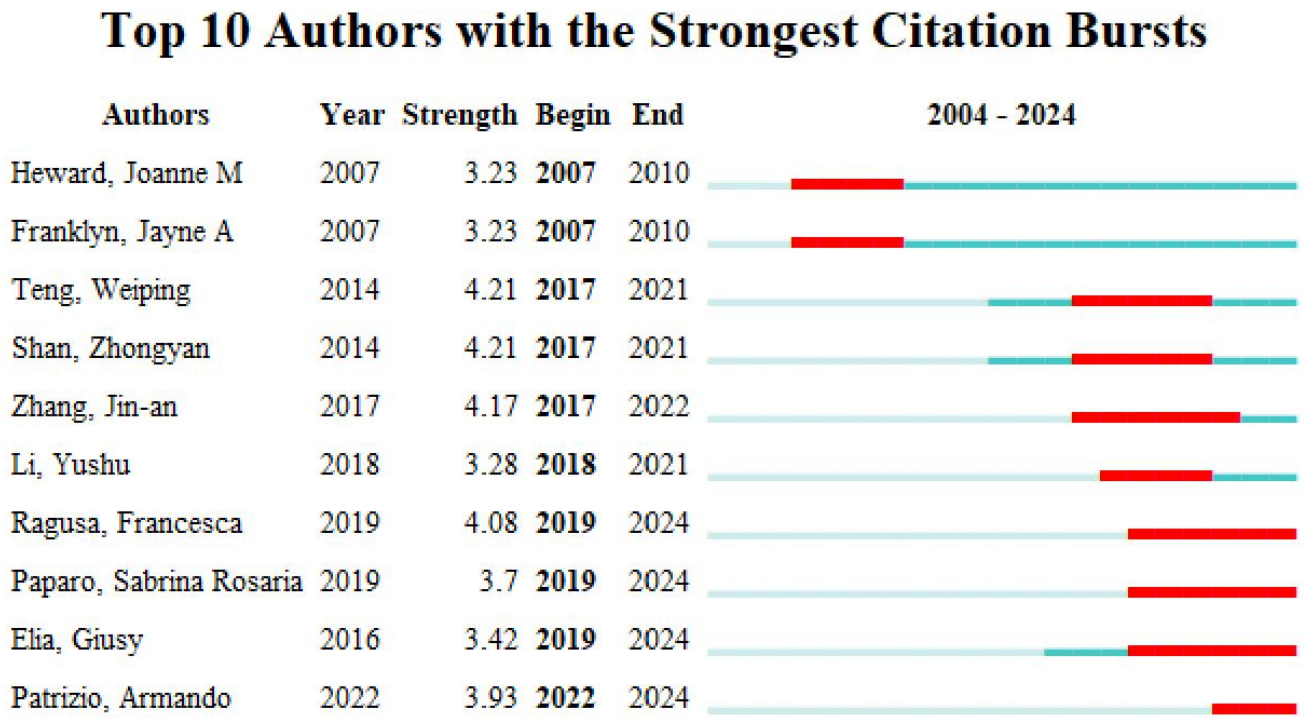

Supplementary Figure 6: Top 10 authors with strong citation bursts. A red bar indicates high citations in that year.

Supplementary Table 1: Top 10 co-cited references on research of AD and AIT.

Rank	Co-cited reference	Citations	Title	Pubmed ID
1	antonelli a, 2015, autoimmun rev, v14, p174, doi 10.1016/j.autrev.2014.10.016	91	Autoimmune thyroid disorders	25461470
2	smith tj, 2016, new engl j med, v375, p1552, doi 10.1056/nejmra1510030	78	Graves' Disease	27797318
3	caturegli p, 2014, autoimmun rev, v13, p391, doi 10.1016/j.autrev.2014.01.007	74	Hashimoto thyroiditis: clinical and diagnostic criteria	24434360
4	ueda h, 2003, nature, v423, p506, doi 10.1038/nature01621	67	Association of the T-cell regulatory gene CTLA4 with susceptibility to autoimmune disease	12724780
5	jacobson dl, 1997, clin immunol immunop, v84, p223, doi 10.1006/clin.1997.4412	65	Epidemiology and estimated population burden of selected autoimmune diseases in the United States	9281381
6	vanderpump mpj, 1995, clin endocrinol, v43, p55, doi 10.1111/j.1365-2265.1995.tb01894.x	56	The incidence of thyroid disorders in the community: a twenty-year follow-up of the Whickham Survey	7641412
7	weetman ap, 2000, new engl j med, v343, p1236, doi 10.1056/nejm200010263431707	55	Graves' disease	11071676
8	bahn rs, 2010, new engl j med, v362, p726, doi 10.1056/nejmra0905750	53	Graves' ophthalmopathy	20181974
9	mcleod dsa, 2012, endocrine, v42, p252, doi 10.1007/s12020-012-9703-2	50	The incidence and prevalence of thyroid autoimmunity	22644837
10	tomer y, 2003, endocr rev, v24, p694, doi 10.1210/er.2002-0030	49	Searching for the autoimmune thyroid disease susceptibility genes: from gene mapping to gene function	14570752

Supplementary Table 2: Top 50 keywords on research of AD and AIT.

Rank	Keywords	Counts	Rank	Keywords	Counts
**1**	Graves Disease	484	**26**	Pregnancy	21
**2**	Autoimmune Disease	474	**27**	COVID-19	20
**3**	Autoimmune Thyroiditis (AIT)	390	**28**	Vitamin D	20
**4**	Hashimoto's Thyroiditis (HT)	236	**29**	Prevalence	19
**5**	Autoimmune Thyroid Disease (AITD)	151	**30**	Systemic Sclerosis	18
**6**	Type 1 Diabetes (T1D)	110	**31**	CTLA-4	17
**7**	Rheumatoid Arthritis (RA)	81	**32**	Inflammation	17
**8**	Single Nucleotide Polymorphism (SNP)	80	**33**	PTPN22	17
**9**	Systemic Lupus Erythematosus (SLE)	79	**34**	Apoptosis	16
**10**	Hyperthyroidism	64	**35**	Thyroglobulin	16
**11**	Celiac Disease	60	**36**	Thyrotoxicosis	16
**12**	Autoantibodies	59	**37**	Gut Microbiota	15
**13**	Graves' Ophthalmopathy	53	**38**	Regulatory T Cells	15
**14**	Hypothyroidism	48	**39**	Antiphospholipid Syndrome	14
**15**	Multiple Sclerosis	46	**40**	Biomarkers	14
**16**	Children	39	**41**	Graves' Orbitopathy	14
**17**	Sjogren's Syndrome	37	**42**	SARS-CoV-2	14
**18**	Cytokines	36	**43**	Antibodies	13
**19**	Human Leukocyte Antigen	31	**44**	Autoimmune Hepatitis	13
**20**	Hashimoto's Disease	27	**45**	Comorbidity	13
**21**	Vitiligo	27	**46**	Iodine	13
**22**	Thyroid Diseases	26	**47**	Polyautoimmunity	13
**23**	Myasthenia Gravis	22	**48**	Thyroid Autoantibodies	13
**24**	Thyroid Cancer	22	**49**	Thyroid Hormones	13
**25**	Addison's Disease	21	**50**	Thyroid Peroxidase	13
